# Supplementary material for: Germinal Center B Cells Replace Their Antigen Receptors in Dark Zones and Fail Light Zone Entry when Immunoglobulin Gene Mutations are Damaging
Source: Immunity. 2018 Sep 18;49(3):477–489.e7. doi: 10.1016/j.immuni.2018.08.025 (PMC6162340; doi:10.1016/j.immuni.2018.08.025)
Supplement: Document S1. Figures S1–S5 [file mmc1.pdf]

Immunity, Volume *49*

Supplemental Information

Germinal Center B Cells Replace Their Antigen Receptors in Dark Zones and Fail Light Zone Entry when Immunoglobulin Gene Mutations are Damaging

Isabelle Stewart, Daniel Radtke, Bethan Phillips, Simon J. McGowan, and Oliver Bannard

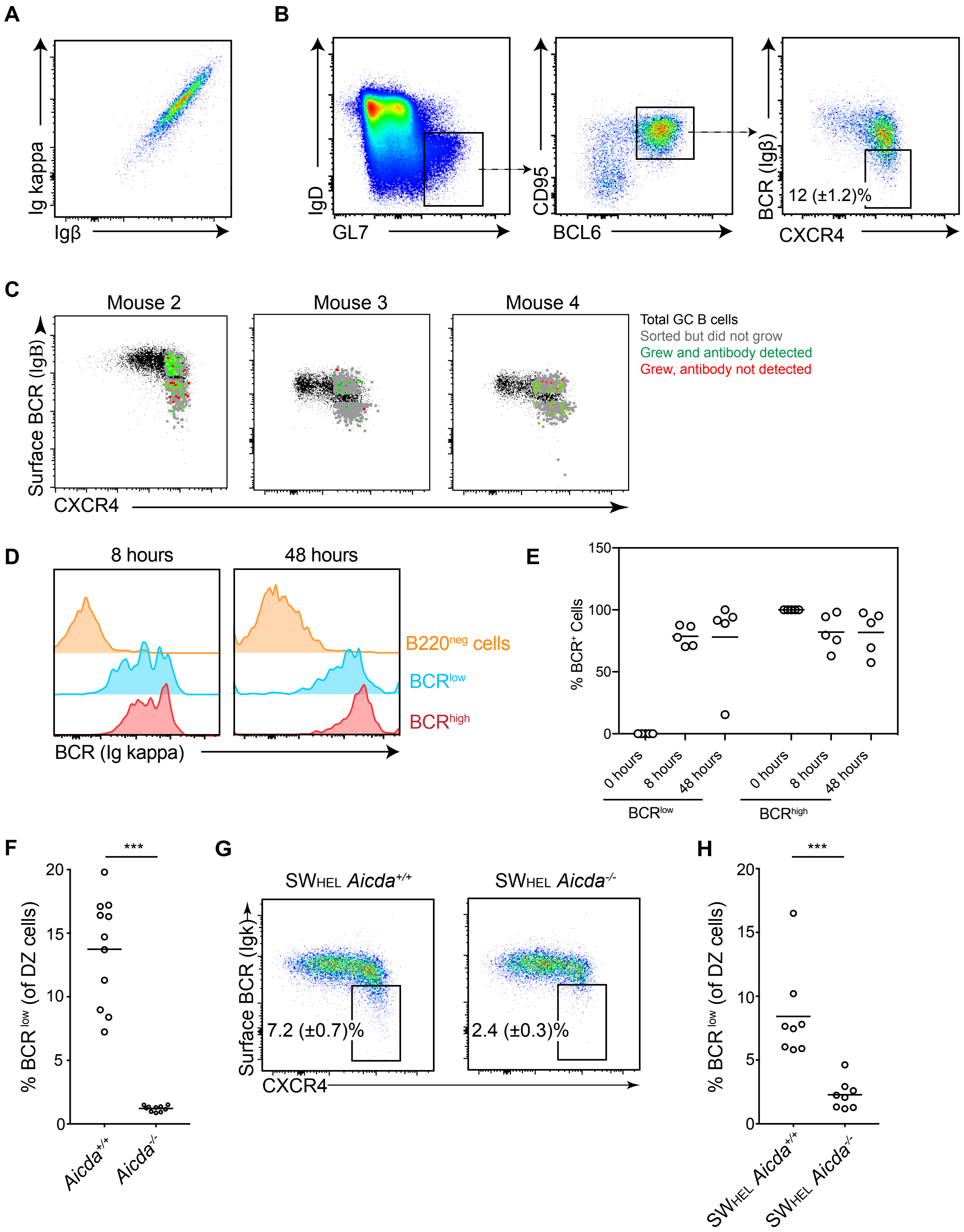
A
Ig kappa
Igβ
B
IgD
GL7
CD95
BCL6
BCR (Igβ)
CXCR4
12 (±1.2)%
C
Mouse 2
Mouse 3
Mouse 4
Surface BCR (IgB)
CXCR4
Total GC B cells
Sorted but did not grow
Grew and antibody detected
Grew, antibody not detected
D
8 hours
48 hours
B220neg cells
BCRlow
BCRhigh
BCR (Ig kappa)
E
% BCR+ Cells
150
100
50
0
0 hours
8 hours
48 hours
0 hours
8 hours
48 hours
BCRlow
BCRhigh
F
***
% BCR low (of DZ cells)
20
15
10
5
0
Aicda+/+
Aicda-/-
G
SWHEL Aicda+/+
SWHEL Aicda-/-
Surface BCR (Igk)
7.2 (±0.7)%
2.4 (±0.3)%
CXCR4
H
***
% BCR low (of DZ cells)
20
15
10
5
0
SWHEL Aicda+/+
SWHEL Aicda-/-

Figure S1 (Related to Figure 1). Characterization of BCR^{low} DZ cells. (A) Surface Igβ and Igκ light chain levels correlate in IgD^{low} CD95^{+} GL7^{+} SWHEL GC B cells (day 7 HEL-OVA/adjuvant immunisation). (B) The CXCR4/BCR staining pattern on BCL6^{+} GC B cells, gated as shown. (C) Results from individual repeat experiments, related to Figures 1F and 1G. Dot plots show BCR and CXCR4 levels on GC B cells at the time of sorting. (D) CXCR4^{high} BCR^{high} and CXCR4^{high} BCR^{low} GC B cells from SRBC immunised mice were sorted for 8 hrs alone, or for 48 hrs under "Nojima" conditions. BCR levels at the end of culture were determined by FACS and compared to that of non-B cells, with data summarised in (E). Labels indicate phenotype at time of sort/plating. (F) Frequencies of *Aicda*$^{+/+}$ and *Aicda*$^{-/-}$ CXCR4^{high} CD83^{low} DZ GC B cells that have low BCR levels in mixed BM chimeric mice on days 7 or 8 of response to SRBC immunisation, related to Figure 1H. (G), (H), Similar assessments were made for co-transferred *Aicda*$^{+/+}$ and *Aicda*$^{-/-}$ SWHEL GC B cells. (A), (B) show representative plot of 7 and 6 mice from 2 experiments, (D) is representative and (E) pooled from 5 mice in 2 experiments, (F) is pooled from 3 experiments and (G)-(H) are from 2 experiments. Numbers in (B), (G) represent mean +/- S.E.M., dots in (E), (F), (H) represent single mice. Analysis was performed using an unpaired two-tailed student's t-test, ***, $P < 0.001$.

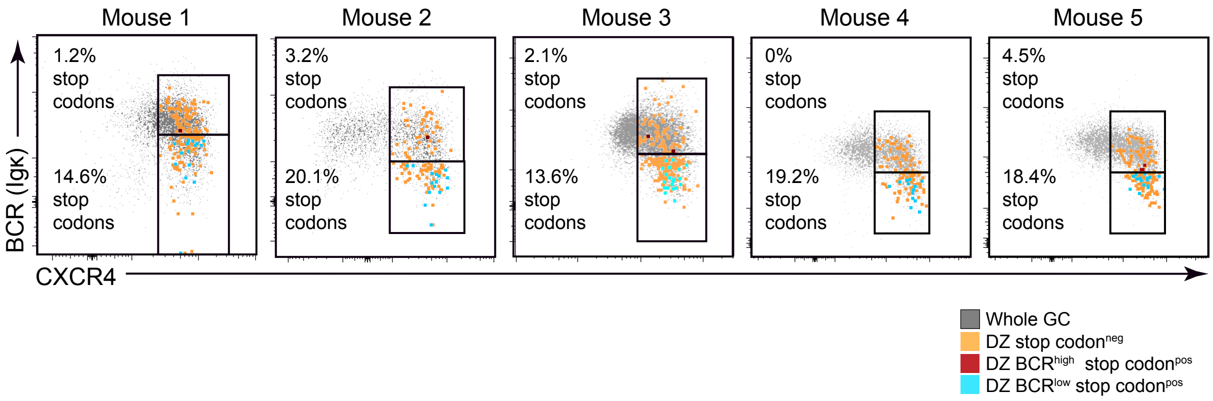
Mouse 1
1.2% stop codons
14.6% stop codons
Mouse 2
3.2% stop codons
20.1% stop codons
Mouse 3
2.1% stop codons
13.6% stop codons
Mouse 4
0% stop codons
19.2% stop codons
Mouse 5
4.5% stop codons
18.4% stop codons
BCR (Igκ)
CXCR4
Whole GC
DZ stop codon neg
DZ BCR high stop codon pos
DZ BCR low stop codon pos

Figure S2 (Related to Figure 2). Cells with premature stop codons accumulate in BCR^{low} DZ subsets. Individual repeats of indexed sequenced cells summarised in Figure 2C (with mouse 3 being the same animal as in Figure 2B). Colours indicate presence or absence of *Ighv* region premature stop codons in cells of the indicated phenotype. Frequencies of premature stop codons in BCR^{high} and BCR^{low} DZ gates are shown.

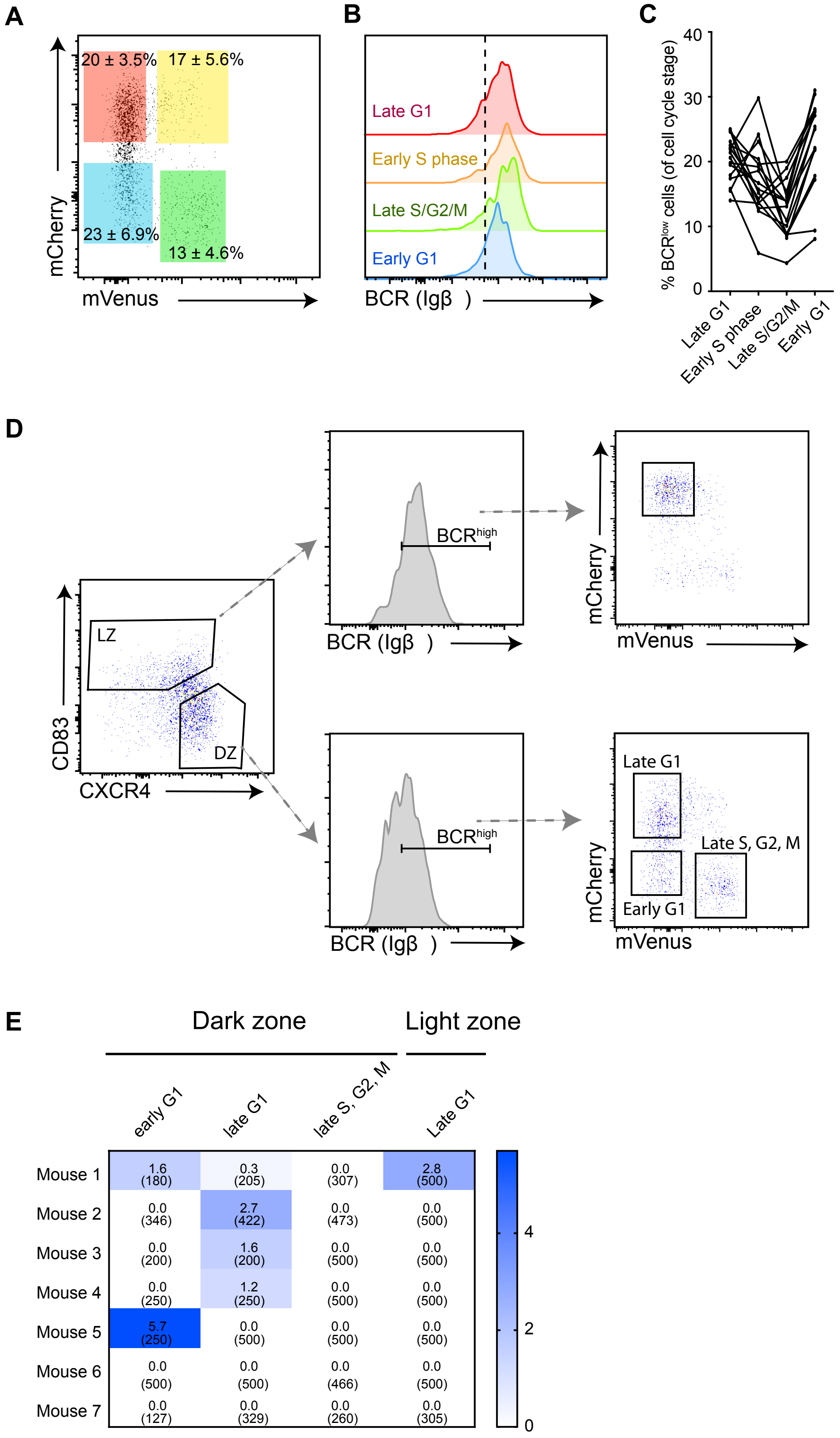
A
20 ± 3.5%
17 ± 5.6%
23 ± 6.9%
13 ± 4.6%
mCherry
mVenus
B
Late G1
Early S phase
Late S/G2/M
Early G1
BCR (Igβ)
C
% BCR^low cells (of cell cycle stage)
40
30
20
10
0
Late G1
Early S phase
Late S/G2/M
Early G1
D
LZ
DZ
CD83
CXCR4
BCR^high
BCR (Igβ)
mCherry
mVenus
Late G1
Late S, G2, M
Early G1
E
Dark zone
Light zone
early G1
late G1
late S, G2, M
Late G1
Mouse 1
1.6 (180)
0.3 (205)
0.0 (307)
2.8 (500)
Mouse 2
0.0 (346)
2.7 (422)
0.0 (473)
0.0 (500)
Mouse 3
0.0 (200)
1.6 (200)
0.0 (500)
0.0 (500)
Mouse 4
0.0 (250)
1.2 (250)
0.0 (500)
0.0 (500)
Mouse 5
5.7 (250)
0.0 (500)
0.0 (500)
0.0 (500)
Mouse 6
0.0 (500)
0.0 (500)
0.0 (466)
0.0 (500)
Mouse 7
0.0 (127)
0.0 (329)
0.0 (260)
0.0 (305)
4
2
0

Figure S3 (Related to Figure 3). BCR^{low} cells are present at all cell cycle stages in the DZ but SHM may occur preferentially in G1. The BCR^{low} state is not restricted to certain cell cycle stages in the dark zone. SWHELxFucci2 DZ (CXCR4^{high} CD83^{low}) GC B cells (IgD^{low} GL7^{+} CD45.1 or 2^{+} or CD95^{+} GL7^{+} CD45.1 or 2^{+}) 8 days after immunisation with HEL-OVA/adjuvant were gated for their cell cycle status (A) and BCR levels determined (B). Dashed line marks BCR^{low} gating. Summary of results from multiple mice and experiments are shown in (C) with each line of connected dots representing results from a single animal. (D) Representative gates used to sort BCR^{high} cells at different cell cycle stages for bulk population based NGS analysis of stop codon presence. (E) The frequency of stop codons detected within each population is shown, with the lower numbers in brackets indicating population sizes sorted from each gate and mouse. Numbers in (A) are means +/- S.D., and (B) is representative of, 13 mice. Six *Aicda*$^{-/-}$ control populations were included in (E) and showed the expected patterns of zero stop codons (not shown).

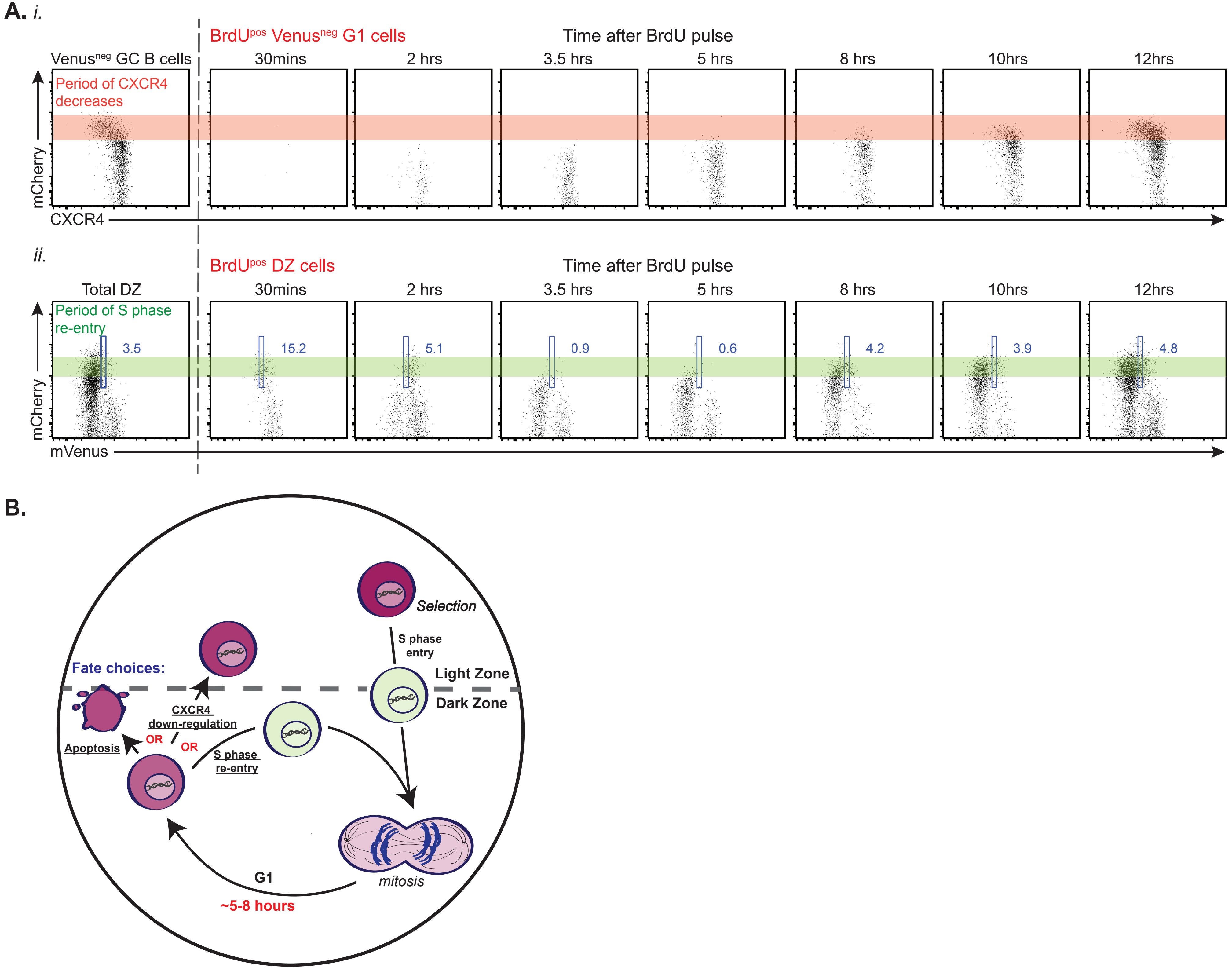

A. i.
BrdU^pos Venus^neg G1 cells
Time after BrdU pulse
Venus^neg GC B cells
30mins
2 hrs
3.5 hrs
5 hrs
8 hrs
10hrs
12hrs
Period of CXCR4 decreases
mCherry
CXCR4
ii.
BrdU^pos DZ cells
Time after BrdU pulse
Total DZ
Period of S phase re-entry
3.5
15.2
5.1
0.9
0.6
4.2
3.9
4.8
mCherry
mVenus
B.
Selection
S phase entry
Light Zone
Dark Zone
Fate choices:
CXCR4 down-regulation
OR
OR
Apoptosis
S phase re-entry
mitosis
G1
~5-8 hours

Figure S4 (Related to Figure 5). The temporal relationship between G1 sub-stage and S phase re-entry, or CXCR4 reductions, by DZ cells. (A) Fucci2 mice were immunised with SRBCs and received single BrdU injections at the indicated time points before analysis on day 10. Plots to left of dash show whole populations irrespective of whether they are $BrdU^{+}$ or $BrdU^{neg}$, plots to right of dash show just $BrdU^{+}$ cells. (i) mVenus (G1) GC B cells were gated to determine the timing of surface CXCR4 decreases after the last cellular division. (ii) $CXCR4^{high}$ $CD83^{low}$ DZ cells were gated. Orange (i) and green (ii) shaded areas indicate ranges of mCherry at which CXCR4 level reductions or S phase re-entry occurs. Very early S phase DZ cells are gated (blue rectangles) in (ii). (B) Cartoon illustrating fate decisions in the GC. "Selected" cells enter S phase in the LZ and complete this first division in the DZ before spending a period of approximately 5-8 hrs in G1. Late G1 DZ cells commit to one of three fates; initiation of another cell division, reduce their CXCR4 levels in preparation for migration to the LZ, or death by apoptosis. Data in (A) are representative of 4-5 mice per time point from 2 independent experiments.

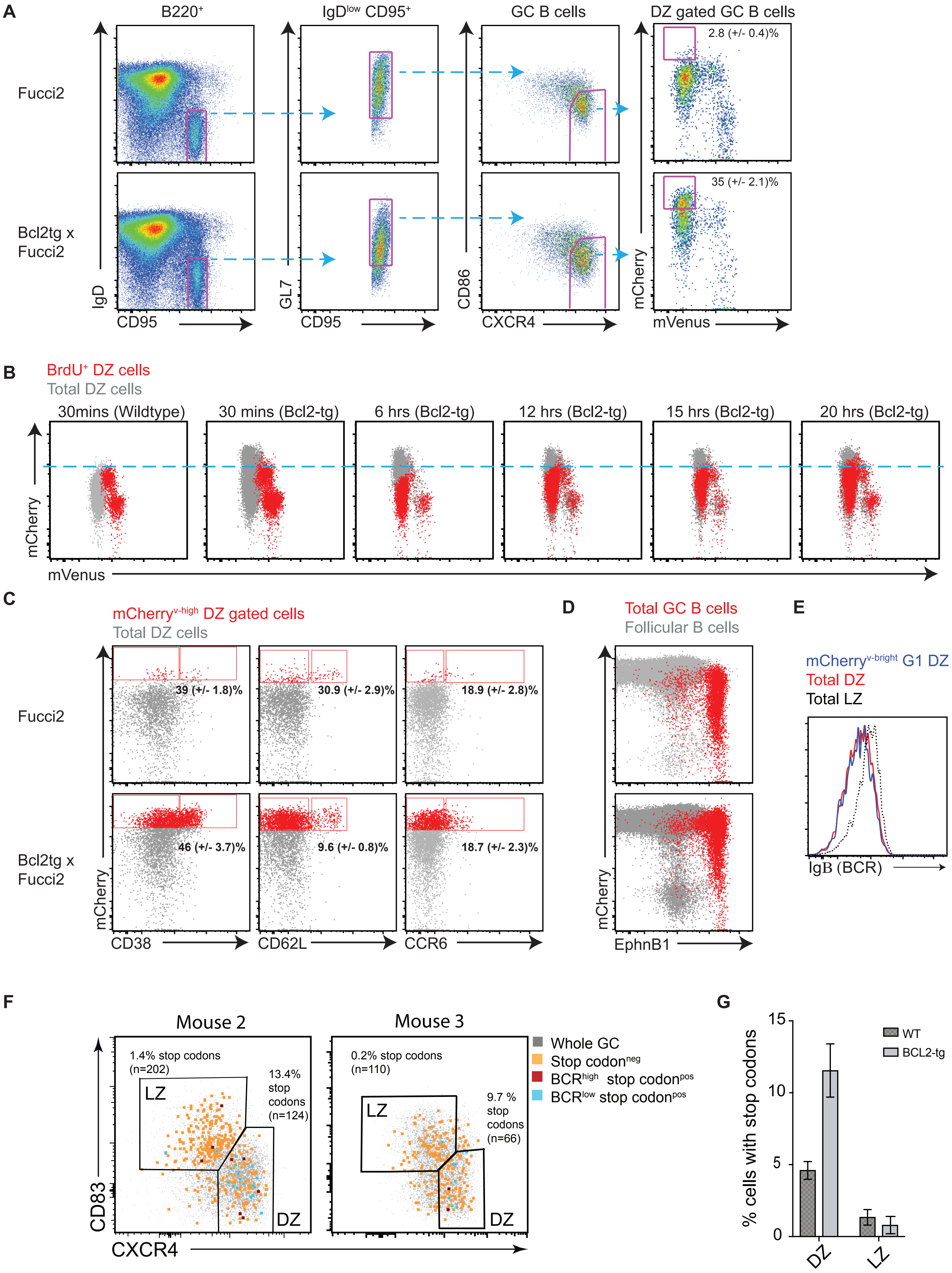
A
B220+
IgDlow CD95+
GC B cells
DZ gated GC B cells
Fucci2
Bcl2tg x Fucci2
2.8 (+/- 0.4)%
35 (+/- 2.1)%
IgD
CD95
GL7
CD95
CD86
CXCR4
mCherry
mVenus
B
BrdU+ DZ cells
Total DZ cells
30mins (Wildtype)
30 mins (Bcl2-tg)
6 hrs (Bcl2-tg)
12 hrs (Bcl2-tg)
15 hrs (Bcl2-tg)
20 hrs (Bcl2-tg)
mCherry
mVenus
C
mCherryv-high DZ gated cells
Total DZ cells
Fucci2
Bcl2tg x Fucci2
39 (+/- 1.8)%
30.9 (+/- 2.9)%
18.9 (+/- 2.8)%
46 (+/- 3.7)%
9.6 (+/- 0.8)%
18.7 (+/- 2.3)%
mCherry
CD38
CD62L
CCR6
D
Total GC B cells
Follicular B cells
mCherry
EphnB1
E
mCherryv-bright G1 DZ
Total DZ
Total LZ
IgB (BCR)
F
Mouse 2
Mouse 3
1.4% stop codons (n=202)
13.4% stop codons (n=124)
0.2% stop codons (n=110)
9.7 % stop codons (n=66)
LZ
DZ
Whole GC
Stop codonneg
BCRhigh stop codonpos
BCRlow stop codonpos
CD83
CXCR4
G
% cells with stop codons
WT
BCL2-tg
0
5
10
15
DZ
LZ

Figure S5 (Related to Figure 6). Aberrant quiescent populations exist in the DZs of Bcl2-tg mice. Splenic GC B cells from SRBC immunised wildtype or Bcl2-tg mice carrying the Fucci2 allele were gated and mCherry levels within DZ cells were determined (A). (B) Mice received single BrdU injections at the indicated time points before analysis. $BrdU^{+}$ DZ cells (red) are overlaid with total DZ cells (grey). The blue dashed bar (equivalent to maximal mCherry levels in WT DZ cells) and is shown for reference. Levels of the various pre-memory markers (C), and the GC and pre-memory associated receptor Ephrin B1 (D), were determined in $mCherry^{v\text{-}bright}$ DZ cells (red) and were overlaid with the total DZ population for reference (grey). Numbers indicate frequency of $mCherry^{v\text{-}bright}$ $mVenus^{neg}$ (red) cells that were positive for indicated protein. (E) BCR levels in GC B populations ($CD38^{low}$ IgD^{low} $GL7^{+}$) of the indicated phenotype. GC DZ/LZ cells were gated using CXCR4 and CD83 for this experiment. (F) Individual repeats of indexed sequenced SWHEL x Bcl2-tg cells that are summarised in Figure 6E. Colours indicate presence or absence of premature stop codons in cells of the indicated phenotype. Frequencies of premature stop codons in DZ and LZ gates are shown, with numbers of cells sequenced shown in brackets. (G) Summary of frequency of stop codons in DZ and LZ of WT (from Figure 4, n=5) and BCL2-tg (n=2) SWHEL GC B cells. Error bars, S.E.M.. Numbers in (A, C, D) are means (+/- S.E.M.) of 6 (WT) and 10 (Bcl2-tg) mice from 2 experiments. Each plot in (B) is from representative of 3-4 mice at each time point from 2 experiments and plots in (E) are representative from 3 experiments each containing 2-3 mice.
