# Supplementary material for: Germinal Center B Cells Replace Their Antigen Receptors in Dark Zones and Fail Light Zone Entry when Immunoglobulin Gene Mutations are Damaging
Source: Immunity. 2018 Sep 18;49(3):477–489.e7. doi: 10.1016/j.immuni.2018.08.025 (PMC6162340; doi:10.1016/j.immuni.2018.08.025)
Supplement: Document S2. Article plus Supplemental Information [file mmc2.pdf]

Immunity

Article

Germinal Center B Cells Replace Their Antigen Receptors in Dark Zones and Fail Light Zone Entry when Immunoglobulin Gene Mutations are Damaging

Graphical Abstract

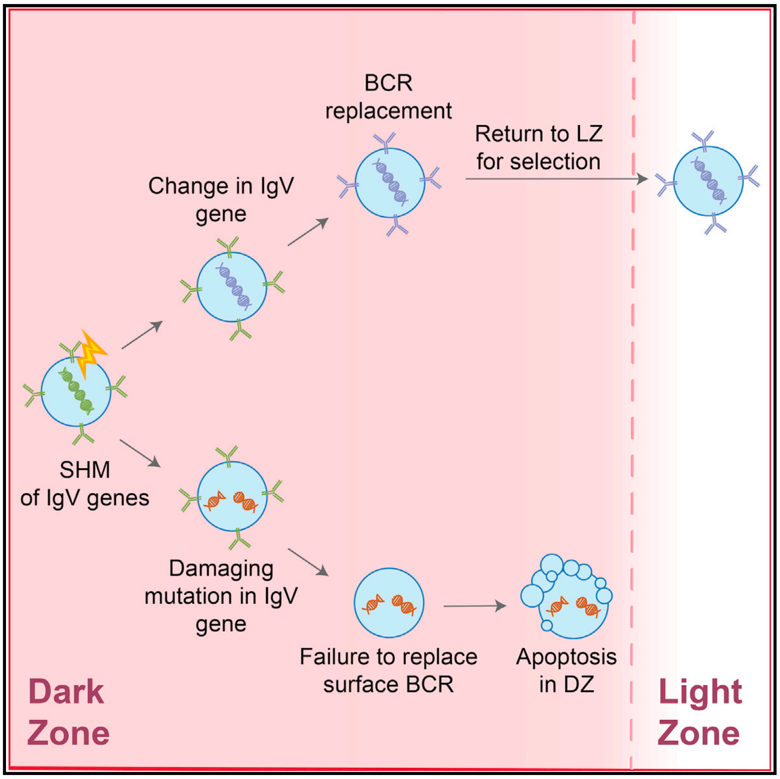

Authors

Isabelle Stewart, Daniel Radtke, Bethan Phillips, Simon J. McGowan, Oliver Bannard

Correspondence

oliver.bannard@ndm.ox.ac.uk

In Brief

Somatic hypermutation is important for the generation of high-affinity antibodies, but this mutational process is also likely to negatively impact the functional integrity of B cell receptors (BCRs). Stewart et al. find that germinal center B cells replace surface BCRs in dark zones (DZ) and present evidence for a DZ checkpoint that prevents the accumulation of clones with non-functional BCRs, thus facilitating selection in the LZ.

Highlights

- GC B cells exchange surface BCRs in dark zones (DZ) following somatic hypermutation
- GC B cells acquiring damaging BCR mutations genes rarely transition to the light zone (LZ)
- Apoptosis of GC B cells in DZ occurs preferentially in the late G1 stage of cell cycle
- Ectopic expression of *Bcl2* fails to rescue LZ entry by B cells with damaged BCRs

Stewart et al., 2018, Immunity *49*, 477–489
September 18, 2018 © 2018 The Author(s). Published by Elsevier Inc.
https://doi.org/10.1016/j.immuni.2018.08.025

Immunity
Article

Germinal Center B Cells Replace Their Antigen Receptors in Dark Zones and Fail Light Zone Entry when Immunoglobulin Gene Mutations are Damaging

Isabelle Stewart,[1] Daniel Radtke,[1] Bethan Phillips,[1] Simon J. McGowan,[2] and Oliver Bannard[1,3,*]
[1]MRC Human Immunology Unit, MRC Weatherall Institute of Molecular Medicine
[2]Computational Biology Research Group, MRC Weatherall Institute of Molecular Medicinex
University of Oxford, Oxford, OX3 9DS, UK
[3]Lead contact
*Correspondence: oliver.bannard@ndm.ox.ac.uk
https://doi.org/10.1016/j.immuni.2018.08.025

SUMMARY

Adaptive immunity involves the development of bespoke antibodies in germinal centers (GCs) through immunoglobulin somatic hypermutation (SHM) in GC dark zones (DZs) and clonal selection in light zones (LZs). Accurate selection requires that cells fully replace surface B cell receptors (BCRs) following SHM, but whether this happens before LZ entry is not clear. We found that most GC B cells degrade pre-SHM receptors before leaving the DZ, and that B cells acquiring crippling mutations during SHM rarely reached the LZ. Instead, apoptosis was triggered preferentially in late G1, a stage wherein cells with functional BCRs re-entered cell cycle or reduced surface expression of the chemokine receptor CXCR4 to enable LZ migration. Ectopic expression of the anti-apoptotic gene *Bcl2* was not sufficient for cells with damaging mutations to reach the LZ, suggesting that BCR-dependent cues may actively facilitate the transition. Thus, BCR replacement and pre-screening in DZs prevents the accumulation of clones with non-functional receptors and facilitates selection in the LZ.

INTRODUCTION

The affinity and breadth of antibodies improves over the course of immune responses as a consequence of antibody affinity maturation in germinal centers (GCs) (Bannard and Cyster, 2017; Mesin et al., 2016). GCs are specialized transient structures that form within B cell follicles in the days following an infection or immunization and can persist for periods of a few of weeks to many months depending upon the nature of the challenge. GCs are anatomically divided into two distinct zones known as light zones (LZs) and dark zones (DZs), with the former forming proximal to the site of antigen entry. LZs are clearly distinguishable by the presence of specialized stromal cells known as follicular dendritic cells (FDCs), which express very high amounts of Fc and complement receptors that sequester antigen-containing immune complexes. DZs are identified by the relative absence of FDCs and by the higher density of proliferating GC B cells.

Antibody affinity enhancements occur through iterative rounds of immunoglobulin variable region (IgV) gene somatic hypermutation (SHM) and selection involving GC B cells transitioning back and forth between DZs and LZs. Movement of GC B cells between the two zones is associated with changes in behavior and phenotype (Allen et al., 2004; Bannard et al., 2013; Victora et al., 2010). Expression of *Aicda* (Muramatsu et al., 2000; Victora et al., 2010; 2012), the gene encoding activation induced cytidine deaminase (AID), is higher in DZ GC B cells. AID catalyzes the deamination of cytosines to uracils, which in turn recruits various error prone repair pathways that cause nucleotide substitutions, additions, or deletions. Mutations in IgV genes are introduced at frequencies of approximately 10^{-3} nucleotides/division, approximately 10^{6}-fold above background mutation rates (McKean et al., 1984). Following antibody diversification in DZs, somatically mutated GC B cells reduce surface expression of the chemokine receptor CXCR4 and migrate to LZs, where they test their newly minted receptors through "selection," a competitive process that involves the acquisition of antigen from FDCs and the presentation of processed peptides to T follicular helper (Tfh) cells (Allen et al., 2004; 2007a; 2007b; Batista and Neuberger, 2000; Victora et al., 2010). Rare cells in which somatic mutations cause antibody affinity improvements are thought to be preferentially selected as a result of them internalizing and processing more antigen to limiting numbers of Tfh cells, thereby driving antibody affinity maturation.

It has become increasingly evident that humoral immune responses are not always best served by rapidly expanding the highest affinity B cell clones at the expense of all others (Bannard and Cyster, 2017; Mesin et al., 2016). For example, the unmutated common ancestors of HIV broadly neutralizing antibodies (bnAbs) often are of very low affinities and only acquire neutralizing potential after accumulating a slew of somatic mutations (Kelsoe and Haynes, 2017; West et al., 2014). While HIV bnAbs may represent extreme examples when it comes to mutation loads, simpler antibodies such as those arising during responses to influenza A infections may also mature in a stepwise process (Lingwood et al., 2012).

 © 2018 The Author(s). Published by Elsevier Inc.
This is an open access article under the CC BY license (http://creativecommons.org/licenses/by/4.0/).

Moreover, secondary memory B cell responses to viral variants and re-assortments benefit from antibody diversification in the GC during the primary challenge (Pappas et al., 2014; Purtha et al., 2011). As such, the preferential selection and expansion of high affinity B cells should not come at the expense of retaining breadth. Consistent with such a notion, two recent studies tracked clonal participation in GCs formed following immunization with complex protein antigens and reported that, while affinity enhancements with time were evident, GCs were remarkably permissive to the retention of low to moderate affinity cells (Kuraoka et al., 2016; Tas et al., 2016). Therefore, GC B cell selection might be as much about screening new pools of somatically mutated cells for their ability to still bind antigen as it is about expanding the very best clones (Bannard and Cyster, 2017). This is necessary because the random nature of SHM means that it is far more likely to negatively impact antigen-binding or be harmful to antibody structure than it is to increase affinity.

The dynamics of GC B cell responses have been intensively studied in recent years with direct measurements of cell movement between zones and states revealing that cells undergo on average two cell divisions between each selection event (Gitlin et al., 2015; 2014), inclusive of the one initiated in the LZ, and that approximately half of all DZ cells transition back to the LZ every 4 hr period (Mesin et al., 2016; Victora et al., 2010). Therefore, the period between a cell somatically mutating its immunoglobulin genes and it entering the selection process is probably quite short (Bannard et al., 2016). This raises the question of whether GC B cells actively replace their BCRs during the intervening period so that they are selected while only expressing the ‘‘new’’ mutated variants. When membrane immunoglobulin half-lives have been measured in follicular B cells, they were found to be long, in the range of 20–80 hr (Andersson et al., 1974). While it is easy to rationalize that GC B cells acquiring affinity enhancing mutations can be preferentially selected without need for complete receptor replacement, it is less clear how detrimental or affinity lowering mutations might be screened out, as is required for diversification, without BCR turnover.

Here, we determined the stage at which pre-SHM antigen receptor complexes are degraded in GC B cells by correlating the acquisition of harmful mutations with the presence or absence of membrane BCRs. Within the DZ, although some cells displayed high surface BCR levels despite carrying damaging mutations in their immunoglobulin genes, two thirds of the cells with that mutation type had low to negligible surface BCR expression, indicating that complete receptor turnover had already occurred. GC B cells carrying damaging mutations did not accumulate in LZ populations as might be predicted if newly minted receptors are first tested at that phase. Instead, apoptosis of cells lacking surface BCR complexes was triggered while cells were still in the DZ state. We propose that the turnover of BCR complexes and the testing of antibody functional integrity following SHM in the DZ prevents the accumulation of cells carrying damaging mutations and facilitates accurate selection for antigen-binding ability when GC B cells reach the LZ.

RESULTS

Existence of Dark Zone GC B Cells with Undetectable Surface BCR Expression

The GC DZ is considered to be where most SHM occurs because it is the site of highest AID mRNA and protein abundance and of expression of error prone repair-pathway-associated genes (Victora et al., 2012; 2010). It therefore follows that GC B cells should turnover and replace their BCR proteins before moving from the DZ to the LZ if they are to be accurately selected. With this in mind, we compared the abundance of membrane BCR proteins on DZ and LZ GC B cells to look for indications that the complexes may be handled differently at the two stages. Levels of the BCR subunit Igβ (CD79b) correlated with those of immunoglobulin light chains (Igκ), providing a means to determine BCR levels irrespective of isotypes (Figure S1A). The majority of CXCR4^{low} LZ cells expressed relatively high BCR levels (albeit lower than follicular B cells), however the CXCR4^{high} DZ population contained within it cells with a ∼2 log range of staining intensities (Figures 1A and 1B). BCL6 staining confirmed CXCR4^{high} BCR^{low} cells as being bonafide GC B cells (Figure S1B) and single cell RT-PCR analysis indicated that differences in BCR levels were not associated with fluctuations in the abundance of *Igh*, *Cd79a*, or *Cd79b* mRNAs (data not shown). Interestingly, GC B cells that stained least brightly were essentially indistinguishable from B220$^{negative(neg)}$ non-B cells in terms of their BCR levels, indicating very little or no protein was present (Figure 1A). To assess intracellular immunoglobulin levels, surface epitopes were saturated with a FITC conjugated antibody before fixation, permeabilization, and further staining with a different antibody-conjugate, revealing the presence of a subpopulation of CXCR4^{high} cells that lacked detectable levels of both surface and intracellular BCRs (Figure 1C). Therefore, the lack of surface protein on GC B cells was not caused simply by endocytic recycling but might instead reflect that BCR complexes had been degraded.

Recognition of self antigen causes anergy in follicular B cells and this is associated with reduced surface IgM levels (Goodnow et al., 1988). However, the decreases in BCR levels by GC B cells was not solely the result of cells acquiring self-reactivity because similar populations existed even when BCR specificity was fixed in hen egg lysozyme (HEL) specific MD4 transgenic and SW$_{HEL}$ gene-targeted B cells (Figures 1D and 1E) (Goodnow et al., 1988; Phan et al., 2003). The presence of BCR^{low} cells within MD4 GC B cell populations also raised the possibility that this state might not be caused only by cells acquiring mutations that completely cripple immunoglobulin expression because, although cells from this mouse line can still undergo SHM, they carry multiple copies of the transgene (Goodnow et al., 1988). Consistent with this, BCR^{low} GC B cells were capable of re-expressing surface BCR when cultured *ex vivo* for 8 hr or 48 hr and they could also be driven to secrete antibodies using the ‘‘Nojima’’ method (Figures S1D–1G) (Kuraoka et al., 2016). The frequency of single BCR^{low} DZ cells successfully cloned was approximately half that of BCR^{high} DZ cells, suggesting that this population may contain cells at a mixture of states including at least some that are destined to die (Figure 1G).

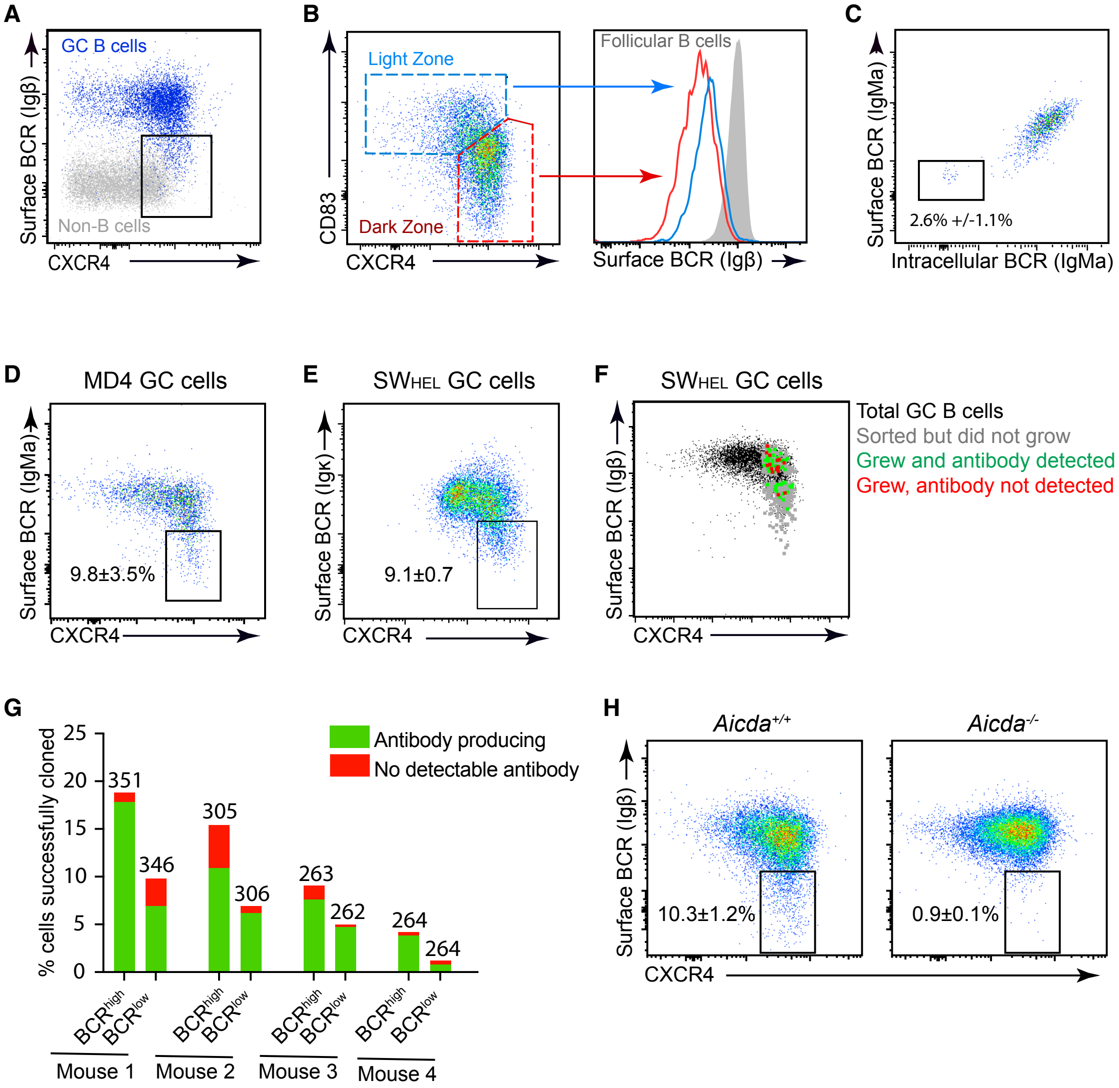

Figure 1. Reduced BCR Levels on DZ GC B Cells

(A and B) Mice were immunized with SRBCs and splenic IgD^{low} CD95^{+} GL7^{+} GC B cells were analyzed on day 7. (A) BCR levels on DZ populations (CXCR4^{high}) were analyzed and compared to B220^{neg} non-B cells. The gate indicates a BCR^{low} DZ population. (B) DZ and LZ cells are gated as shown and BCR levels overlaid with IgD^{+} CD95^{low} follicular B cells.

(C–G) HEL-specific SW$_{HEL}$ or MD4 B cells were adoptively co-transferred with OT-II CD4^{+} T cells and mice were immunized with HEL-OVA/adjuvant adjuvant. Splenic IgD^{low} CD95^{+} GL7^{+} CD45.1^{+} GC B cells were analyzed on day 7. (C) Surface and intracellular BCR (IgMa) levels were co-stained in fixed/permeabilized MD4 GC B cells revealing a subset of cells that is negative for both. (D and E) DZ cells with low BCR levels in MD4 transgenic (D) and SW$_{HEL}$ gene targeted GC B cell populations (E). (F and G) Single CXCR4^{high} BCR^{high} and CXCR4^{high} BCR^{low} DZ SW$_{HEL}$ GC B cells (gated IgD^{low}, CD38^{low}, GL7^{+}, CD45.1^{+}, or CD45.2^{+}) were index sorted and cultured for 8 days on NB21 feeder cells. (F) The BCR and CXCR4 staining profiles (at the time of sort) of single GC B cells that grew and secreted detectable antibody after culture are shown. The plots for cells that did not grow, or that grew did not secrete detected antibody, are also shown and overlaid with the total GC B cell population for reference. Plots for additional mice are shown in Figure S1. (G) Summary of results. Numbers of cells in each group per experiment are indicated.

(H) Surface BCR levels on *Aicda*$^{+/+}$ and *Aicda*$^{-/-}$ IgD^{low} CD95^{+} GL7^{+} GC cells from mixed bone marrow chimeric mice 8 days after SRBC immunisation. Numbers in (C–E) and (H) represent mean ± SEM from 7 (C and D), 10 (E) and 11 (H) mice from 2–4 experiments. See also Figure S1.

The BCR^{low} DZ subset does, however, depend upon the SHM machinery because it was almost absent in AID deficient (*Aicda*$^{-/-}$) GC B cell populations (Figures 1H and S1F–S1H).

In summary, GC DZs contain cells that have very low or undetectable BCR levels that depend upon AID for their normal generation.

GC B Cells Replace Their Antigen Receptors following Somatic Hypermutation but While Still in the Dark Zone

The average period that GC B cells spend in the DZ during each visit is relatively short (Gitlin et al., 2015; Victora et al., 2010). We therefore investigated whether the turnover and replacement of pre-SHM BCRs with newly mutated versions may occur while

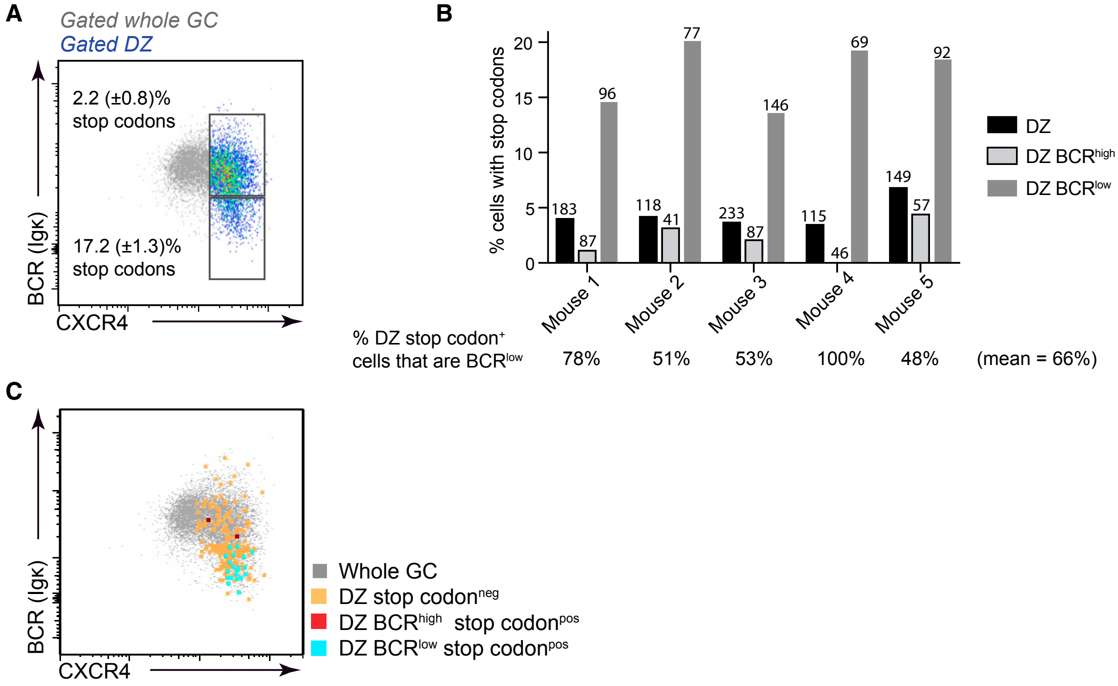

Figure 2. DZ Cells Acquiring Detrimental Mutations in Their Immunoglobulin V Region Genes Accumulate in BCR^{low} Gates

SW$_{HEL}$xFucci2 B cells were co-transferred with OT-II CD4$^+$ T cells into WT hosts that were subsequently immunized with HEL-OVA/adjuvant. Single IgD^{low} CD95$^+$ GL7$^+$ (mice 1-3), or IgD^{low} CD95$^+$ (mice 4 or 5), CD45.1/2$^+$ SW$_{HEL}$ GC B cells were index sorted on day 8 and their *Ighv* region genes were PCR amplified, Sanger sequenced, and analyzed for the presence of premature stop codons.

(A) Frequency (mean ± S.E.M., n = 5) of premature stop codons in BCR^{high} and BCR^{low} CXCR4^{high} CD83^{low} DZ populations is shown.

(B) Summary of results from multiple mice and experiments. Numbers of cells sequenced are indicated above bars and the proportion of total DZ stop codons that are in the BCR^{low} subset is marked below. Results are normalized for rare population enrichments performed during FACS sorting.

(C) BCR and CXCR4 levels on individual indexed sequenced cells. Colors indicate presence or absence of *Ighv* region premature stop codons in cells of the indicated phenotype (pos, positive, neg, negative). Data are overlaid with cells that were not sequenced (gray), for reference. Less frequent BCR^{low} cells were often enriched during FACS sorting. See also Figure S2.

cells are still in that zone. Due to the random nature of SHM, no reagents exist that allow for the identification of individual cells with pre- and post- SHM immunoglobulin proteins on their surface. However, we reasoned that by correlating single cell IgV region gene sequences with surface BCR levels it may be possible to identify cells that have recently acquired harmful mutations and ask whether they still have detectable antigen receptor complexes. The identification of cells carrying damaging mutations but that still have surface BCRs represents a unique opportunity to find cells which are expressing a BCR that they no longer encode at the gene level. As such, it should be possible to determine to what extent BCR proteins are replaced before cells leave the DZ.

Based upon published estimates of SHM rates (10^{-3} nucleotides/division), we calculated that DZ populations should contain stop codons in the heavy chain variable region (*Ighv*) gene at frequencies of ~4.1% when cells complete two cell cycles in that compartment, providing us with confidence that detecting such cells should be feasible (McKean et al., 1984) (calculations in Star Methods). Although premature stop codons are not the only mutation type that will be detrimental to the immunoglobulin protein structure, they provide a relatively easy-to-measure parameter. Single HEL-specific SW$_{HEL}$ GC B cells were index sorted on day 8 following HEL-Ovalbumin (HEL-OVA)/adjuvant immunization and the presence or absence of premature stop codons in their *Ighv* genes was determined by PCR amplification and Sanger sequencing (Phan et al., 2003). The use of gene targeted B cells with a fixed *Ighv* gene allowed for the amplification and sequencing of gDNA sequences, thereby overcoming concerns regarding reverse transcriptase fidelity, non-sense mediated mRNA decay and non-productive allele transcription.

Ighv region stop codons were present in BCR^{high} DZ cells at a frequency of 2.2% but were strikingly enriched in cells with low BCR levels, being > 7-fold more abundant (17.2%) (Figure 2A). Results from multiple experiments are also shown (Figure 2B). As such, ~66% of DZ cells carrying SHM induced stop codons had decreased their surface receptor levels in the period since that event. Premature stop codon encoding mutations were not detected in *Aicda*$^{-/-}$ SW$_{HEL}$ GC B cells (n = 25), confirming the fidelity of the assay (data not shown). The BCR staining intensities of individual cells carrying the damaging mutations are shown in Figure 2C for one experiment (and for all five mice in Figure S2) where they are overlaid with the total GC (for reference) and sequenced DZ B cells. These results highlight that BCR^{low} DZ populations contain post-SHM unselected cells and suggest that GC B cells may turnover their BCR proteins following SHM while still in the DZ.

Evidence of Somatic Hypermutation at G1 in the Dark Zone followed by Continued Initiation of and Progression through Cell Cycle without Functional BCR Genes

Alongside SHM, the other defining cellular process of the GC DZ is cellular division. We therefore extended our analysis to ask whether any correlation existed between cell cycle stage and the phenotypes discussed above. Fucci2 mice provide a useful tool for such questions because they carry a Rosa26 promoter driven bi-directional transgene consisting of mVenus-hGem(1/1 10) and mCherry-hCdt1(30/120) (Abe et al., 2013). mVenus protein begins to accumulate as cells enter S phase and its fluorescence intensity increases with time while mCherry is concurrently degraded. Then, at the completion of mitosis, cells begin to accumulate mCherry again. As such, mVenus fluorescence marks actively dividing cells and the relative abundance of mCherry in mVenus^{neg} cells provides an indication of time since completion of mitosis; it is therefore possible to determine the cell cycle stage and the relative time a cell has been in that phase. The accuracy of the Fucci2 reporter in GC B cells was validated by co-staining for DNA content (Figure 3A) and by confirming that mCherry^{low} mVenus^{neg} post-mitotic "early G1" cells did not incorporate BrdU when the pulse was very short (30 mins) but did if assessed 2 hr later (Figure 3B). LZ cells remain in G1 while they await selection and consequently mCherry levels were higher in cells from that zone (Figure 3C).

BCR^{low} DZ cells were abundant in both S/G2/M and in early G1 cells suggesting that this phenotype is not caused solely by asymmetrical distribution of the receptor complex (Figures S3A–S3C). We analyzed the indexed *Ighv* region sequencing results to determine whether any correlation existed between the presence of disruptive stop codons and cell cycle stage. If BCR turnover following SHM is fast, then BCR^{high} cells with deleterious mutations are those that have very recently undergone SHM. Interestingly, all DZ cells with this phenotype were in G1 phase raising the possibility that SHM may occur preferentially at this stage in GC B cells *in vivo* (Figures 3D and 3E). The rarity of such cells meant that the absolute numbers of BCR^{high} stop codon^{+} events detected at each cell cycle stage was very low, however consistent findings were also made using a bulk population next generation sequencing approach in which cells from 7 additional mice were interrogated (Figures S3D and S3E). We observed slightly lower stop codon frequencies overall using the NGS sequencing method which probably reflected the stringency of the BCR^{high} gates used in these particular experiments. An association of AID activity and G1 was previously reported in *in vitro* B cell culture systems (Le and Maizels, 2015; Wang et al., 2017a). In contrast, BCR^{low} cells carrying premature stop codons were observed at all stages of cell cycle in the DZ (early G1, late G1, early-S, late G2/M) indicating that BCR dependent signals do not control the transitioning between cell cycle phases once in the DZ state (Figures 3D and 3F).

GC B Cells Acquiring Damaging Immunoglobulin Gene Mutations Rarely Reach the Light Zone Phase

Models for antibody affinity maturation involve GC B cells moving to the LZ following SHM in order to "test" their newly minted BCRs via a competitive process involving interactions with follicular helper T cells (Allen et al., 2007a; Victora and Nussenzweig, 2012). Cells that have acquired affinity conferring mutations have an increased likelihood of passing the bottleneck while cells now carrying detrimental mutations will fail selection and die by neglect (Liu et al., 1989). If this were the only time where functional BCRs were needed, then this model would predict that GC B cells carrying detrimental mutations should accumulate in the LZ as they await selection. To test this, the frequency of cells with *Ighv* premature stop codons within LZ and DZ populations was compared. Detrimental mutations were present in the CXCR4^{high} CD83^{low} DZ population at approximately three and a half fold the frequency to within the CXCR4^{low} CD83^{high} LZ (4.6% versus 1.3%) (Figures 4A and 4B). Where LZ cells were stop codon^{+}, they were more commonly BCR^{high} (Figure 4C), indicating either that cells infrequently but occasionally transition to the LZ before fully replacing their BCRs or that they sometimes undergo SHM while in that zone. These results do not fit with the expectation of the models discussed above. Therefore, GC B cells mostly turn over their BCRs in the DZ and do not frequently transition to the LZ stage when can no longer translate a functional receptor complex.

GC B Cells May Undergo Cell Death as an Alternative Fate to Initiating Another Cell Division or Transitioning to the Light Zone Stage

The finding that GC B cells acquiring detrimental IgV region mutations were not commonly found within LZ gates suggested that cells losing expression of a functional antigen receptor may die while still in the DZ. The site and timing of apoptosis in GC was therefore investigated by staining fixed and permeabilized cells with a monoclonal antibody specific for the active form of caspase3 (act-Casp3). Act-Casp3^{+} GC B cells were detected in both LZ and DZ gated populations, however the frequency was slightly higher in the latter subset which is also typically 2-3x larger (Figures 5A and 5B). Therefore, most GC B cells die in the DZ rather than during selection in the LZ.

That GC B cells move to the LZ a number of hours after exiting cell cycle in the DZ is well documented (Allen et al., 2007b). However, an examination of mVenus and mCherry fluorescence patterns in GC B cells from the Fucci2 mice revealed that the timing at which cells reduced their CXCR4 levels was tightly co-ordinated with a specific period in late G1 (Figure 5C). BrdU pulse-chasing experiments revealed that this period corresponded to ~8-10 hr after the completion of the last S phase because it took cells this amount of time after labeling to reach the relevant mCherry levels (Figure S4Ai). By comparison, S phase re-entry in the DZ occurred ~5–8 hr after the previous period of DNA synthesis (Figure S4Aii). As such, cells destined to return to the LZ began reducing their CXCR4 levels at periods that were only slightly later than that of S phase re-entry by DZ cells initiating more cellular divisions. This late G1 period can therefore be considered a fate junction at which cells must be committed to S phase re-entry or to CXCR4 decreases (period corresponding to shaded yellow area on Figure 5C). With this in mind, it was notable to us that in earlier experiments, GC B cells with damaged *Ighv* genes and very low BCR levels had been reasonably abundant at this late G1 phase and in the populations of cell re-entering S phase from it despite their being almost absent from the LZ which is only separated by a short period

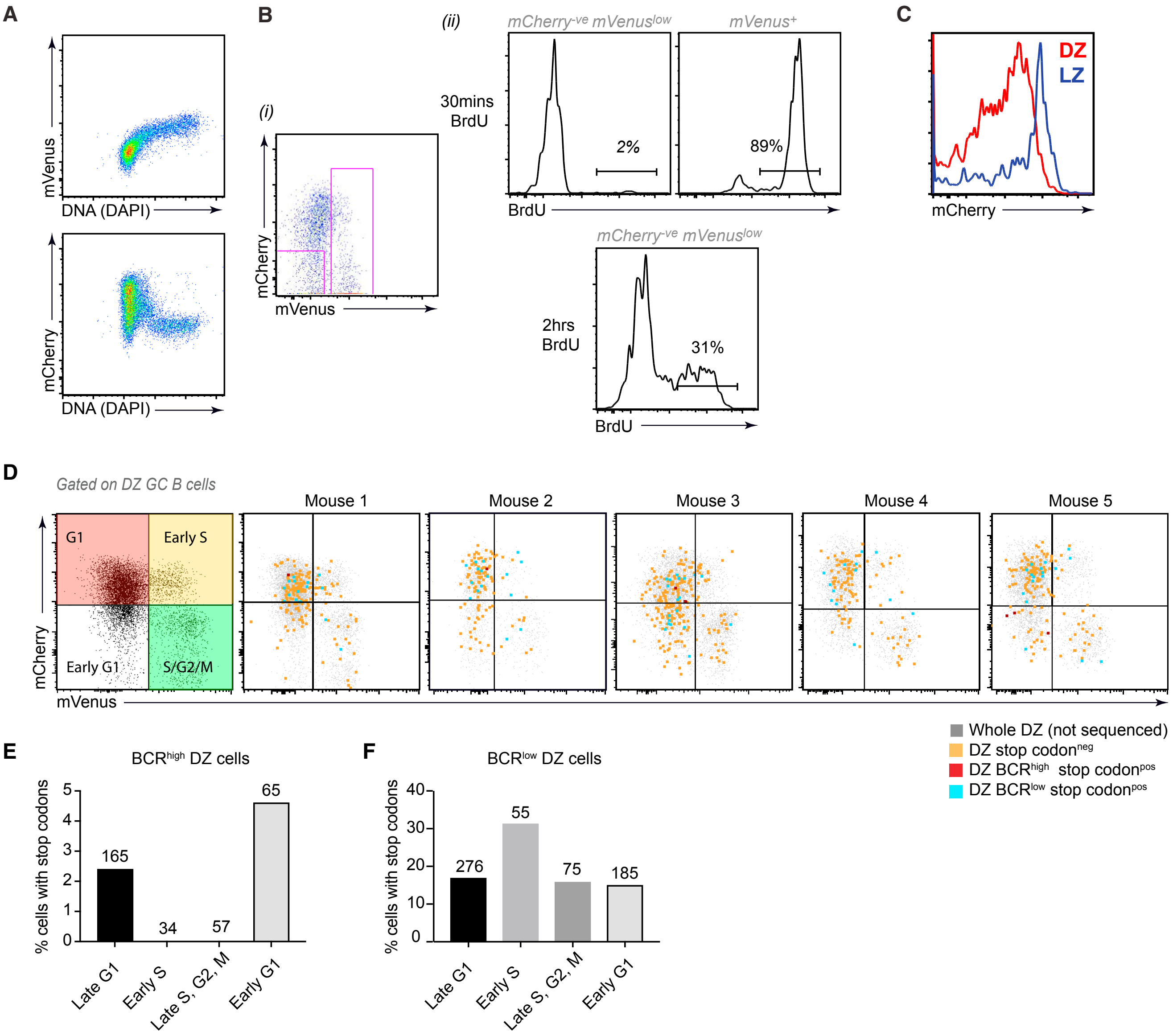

Figure 3. Correlating Cell Cycle and the Acquisition of Stop Codons

(A) Verification of utility of Fucci2 reporter mice for tracking cell cycle in IgD^{low} CD95^{+} GL7^{+} GC B cells on day 10 following SRBC immunisation. mVenus accumulates during S phase as is evidenced by its correlation with DNA content; mCherry is degraded during S phase but accumulates during G1.

(B) S/G2/M (mVenus^{+}) and early G1 (mCherry- mVenus^{low}) CXCR4^{high} CD83^{low} DZ cells were gated (i) and assessed for BrdU incorporation 30 minutes and 2 hours after a single injection (ii). S phase cells label at 30 mins while labeling of early G1 cells require completion of mitosis.

(C) mCherry levels in CXCR4^{low} CD83^{high} LZ and CXCR4^{high} CD83^{low} DZ cells. (D and E) SW$_{HEL}$xFucci2 CXCR4^{high} CD83^{low} DZ cells 8 days after immunization with HEL-OVA/adjuvant (as in Figure 2).

(D) Gates indicate the cell cycle status of DZ SW$_{HEL}$xFucci2 GC B cells (annotated in left plot). Cells with high and low BCR levels that have acquired premature *Ighv* region stop codons, as well as sequenced cells in which stop codons were not detected, are shown (right). Data are overlaid with cells that were not sequenced (gray), for reference.

(E and F) Frequency of stop codons among DZ BCR^{high} (E) and DZ BCR^{low} (F) cells at the different cell-cycle stages. (E) includes results from 3 additional mice from which rare BCR^{low} S and early G1 phase cells were enriched to increased cell numbers. Plots in (A) are representative of 8 mice from 2 experiments, (B) of 4 mice from 2 experiments, (C) of 10 mice from 3 experiments. See also Figure S3.

of time (Figures 3D and 4A). These observations lead us to examine whether any association exists between cell cycle stage and the timing of apoptosis in the DZ.

DZ cells staining for act-Casp3 were present at all stages of cell cycle indicating that apoptosis can occur at any time (Figures 5D). However, we observed an enrichment within the apoptotic gate of cells at late G1 (mCherry^{high} mVenus^{neg}) indicating that a disproportionate amount of cell death occurred at this stage (Figures 5D and 5E). Act-Casp3^{+} populations were highly enriched for cells with low surface BCR levels (Figures 5F and G)

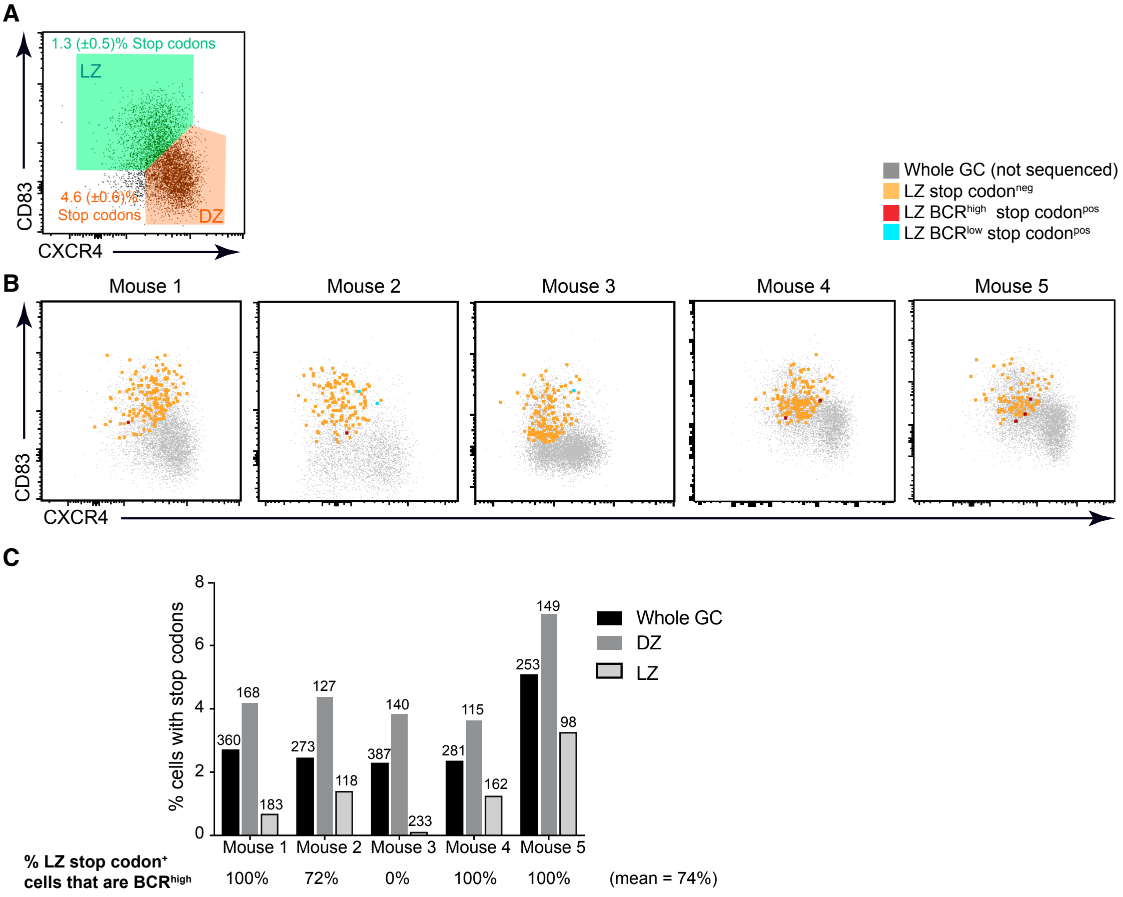

Figure 4. Cells Carrying Premature Stop Codons Fail to Accumulate in the LZ

(A) The frequency of cells carrying *Ighv* region premature stop codons in SW$_{HEL}$xFucci2 LZ and DZ populations was determined. Plot and gate are from a representative mouse, numbers indicating means (+/− SEM) from five independent experiments.

(B) The phenotype of LZ cells with premature stop codons is shown for all three mice. Data are overlaid with total GC B cells that were not sequenced (gray), for reference.

(C) Summary from five independent experiments. Numbers above bars indicate number of each cell type sequenced. Numbers below indicate frequency of detected LZ stop codons that were BCR^{high} and have been normalized for where rare populations were enrichments performed during FACS sorting.

and 60% of apoptotic BCR^{low} DZ cells were at this cell-cycle stage (Figures 5D and E). Therefore, GC B cells may undergo cell death at any time while in the DZ but they are most likely to do so in late G1 when other cells are re-entering S phase or transitioning to the LZ. Consequently, mCherry levels in apoptotic DZ cells were higher than in non-apoptotic CXCR4^{high} CD83^{low} cells but lower than in the LZ (Figure 5H). These results are consistent with earlier findings that the DZ to LZ transition is determined by an intrinsic timer (Bannard et al., 2013), but also suggest that cells may not all be equally competent for the change. Rapid cell death may be the default pathway when cells exhaust their DZ proliferative program but also do not receive, or respond to, LZ promoting cues (Figure S4B).

The findings that GC B cells carrying damaged IgV region genes did not reach the LZ and that cells with low BCR levels preferentially died in late G1 prompted us to investigate whether the delaying or prevention of cell death might be sufficient for GC B cells with detrimental mutations to transition to the LZ state. GCs from mice carrying a B cell specific *Bcl2* anti-apoptotic transgene contained slightly larger DZs and an increased frequency of CXCR4^{high} BCR^{low} cells (Figures 6A and B). Interestingly, when characterizing GCs from Bcl2-tg mice also carrying the Fucci2 allele we noticed that approximately a third of the DZ cells were mCherry$^{very\text{-}high}$ and mVenus^{neg} suggesting that they may be quiescent (Figure S5A). Wild-type DZ cells generally remained in G1 for a maximum of ~8–10 hr (Figure S4A), however the majority of the mCherry$^{v\text{-}bright}$ Bcl2-tg DZ cells were still BrdU^{-ve} even after a 20 hr chase period (Figure S5B). A proportion of the quiescent DZ subset expressed various recently described pre-memory cell markers (CCR6, CD38, CD62L, Ephrin B1) (Figures S5C and S5D) (Laidlaw et al., 2017) (Wang et al., 2017b) (Suan et al., 2017), but their BCR levels were indistinguishable from that of the total DZ population (Figure S5E).

Premature *Ighv* stop codons were present in *Bcl2*-tg^{+} SW$_{HEL}$ GC B cells populations at approximately 2.5-fold that in WT mice and were 45% higher within the BCR^{low} subset than in the WT equivalent (25.0% versus 17.2%), supporting the notion

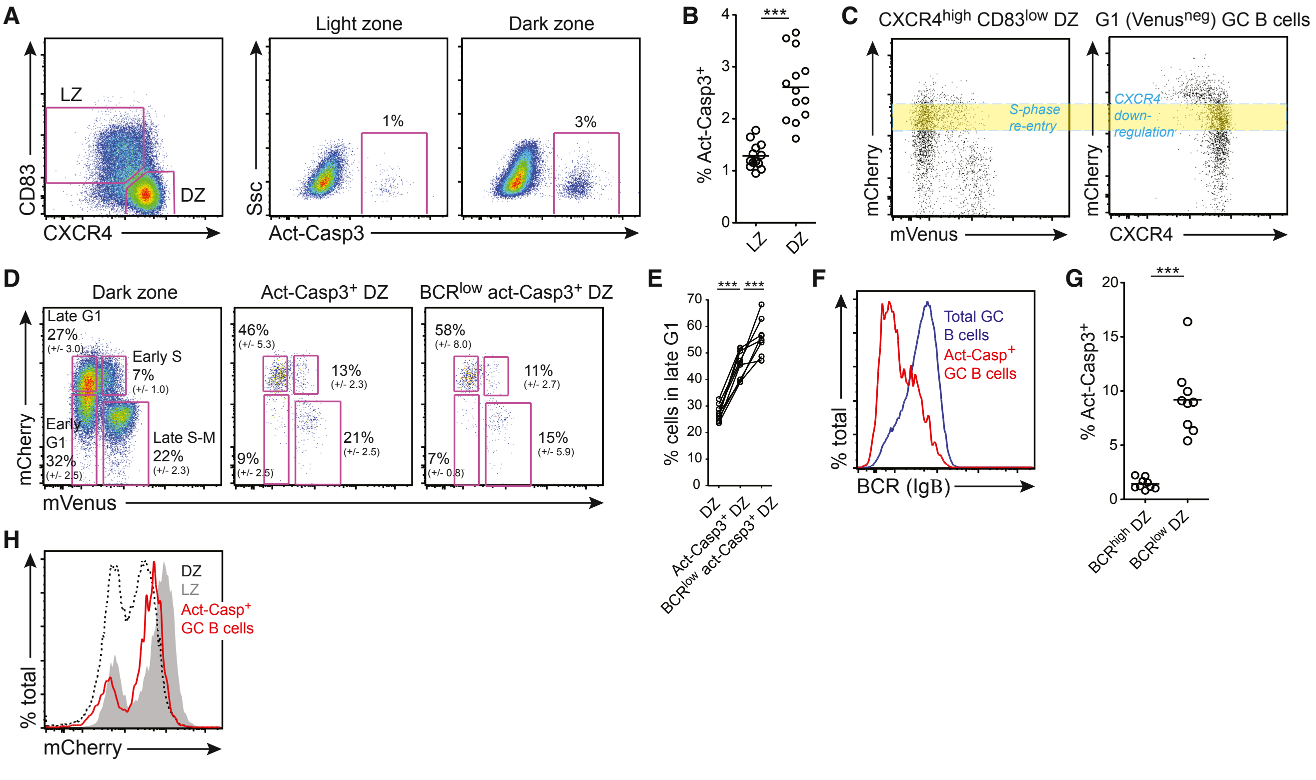

Figure 5. BCR^{low} GC B Cells Undergo Apoptosis during Late G1 in the DZ

Fucci2 mice were immunized with SRBCs and splenic IgD^{low} CD95^{+} GL7^{+} GC B cells were analyzed on day 10. (A and B) GC B cells were stained with LZ/DZ markers and with anti- act-Casp3.

(C) The timing of cell cycle re-entry from the DZ or surface CXCR4 decreases by G1 GC B cells was determined by examining mCherry levels. Yellow area indicates a likely fate junction where cells must be committed one of these processes.

(D and E) DZ, act-Casp3^{+} DZ, and BCR^{low} act-Casp3^{+} DZ cells were gated and levels of the cell cycle regulated Fucci2 florescent reporters were examined. Cell-cycle stages are gated in (D) and mean frequencies ± SD are shown, with summaries in (E).

(F) BCR (Igβ) levels on total GC B cells and act-Casp3^{+} GC B cells.

(G) Frequency of BCR^{high} and BCR^{low} DZ cells that were act-Casp3^{+}.

(H) mCherry levels in LZ, DZ, and Act-Casp3^{+} cells are shown. Plots and graphs in (A–C) are representative of 13 mice from 4 experiments; plots and means (+/− SD) in (D–H) are compiled from, or representative of (F and H), of 9 mice from 3 experiments. Each point in (B, E, G) represents a single mouse. Analysis was performed using an unpaired two-tailed Student's t test. ***$p < 0.001$. See also Figure S4.

that the transgene allows cells to persist for longer after a detrimental SHM event has occurred (Figures 6C and 6D, S5F). Surprisingly, the frequency of detrimental somatic mutations was also increased within the DZ BCR^{high} (but not LZ BCR^{high}) gates, perhaps indicating higher SHM rates in these cells or a general increased resistance to genotoxic stress (Le and Maizels, 2015). The latter might cause the former if AID is active at inappropriate times or cell-cycle stages in Bcl2-tg cells. Despite the striking increases in the DZ subsets (11.6% versus 4.6% for WT DZ cells), the promotion of cell survival through expression of the *Bcl2* transgene was not sufficient to increase the frequency of cells with premature stop codons present within LZ gates (0.8% of CXCR4^{low} CD83^{high} cells in *Bcl2*-transgenic versus 1.3% in WT cells) (Figures 6E, S5G, 4A). While it is possible that SHM and LZ re-entry are temporally separated to a degree that cannot be rescued by the transgene, these results raise the possibility that BCR-dependent cues might be necessary not only for survival in the DZ but also for the transitioning to the LZ state.

DISCUSSION

That GCs are sites of intra-clonal competition that leads to the preferential expansion of high-affinity antibody mutants has been recognized for almost three decades (Berek et al., 1991; Jacob et al., 1991). However, it has become increasingly evident in recent years that these unique structures also play critical roles in diversifying humoral responses (Bannard and Cyster, 2017; Mesin et al., 2016). Consequently, for a competitive environment, the GC can be remarkably permissive to retaining lower affinity clones (Kuraoka et al., 2016; Tas et al., 2016). Neutralizing epitopes on pathogens are often not those which are most abundant or "easy" for antibody binding and important key neutralizing epitopes on some viruses such as HIV share structural similarities to self-antigens (Kelsoe and Haynes, 2017; West et al., 2014). As such, SHM at multiple sites across the IgV region genes may be required for antibodies to properly reach maturity. The conceptual challenges that GC responses face therefore seem to extend beyond screening cells for affinity

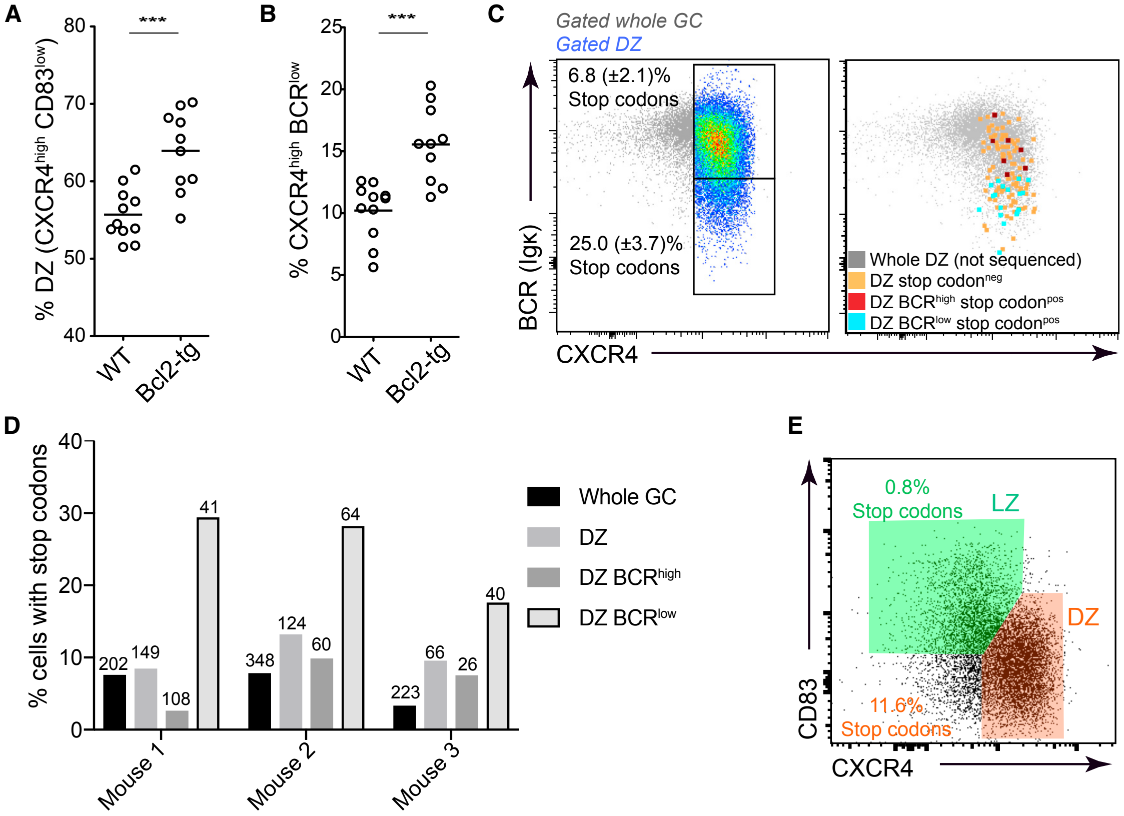

Figure 6. Promotion of Survival Is Not Sufficient to Permit Cells with Non-Functional BCRs to Enter the LZ

(A and B) Fucci2 mice with or without (WT) a BCL2 transgene (BCL2-tg) were immunized with SRBCs and analyzed day 10 (A) Frequencies of CXCR4^{high} CD83^{low} DZ cells and (B) CXCR4^{high} BCR(Igβ)^{low} cells in GCs (CD38^{low} IgD^{low} GL7^{+}).

(C–E) SW$_{HEL}$xBCL2-tg B cells were co-transferred with OT-II CD4^{+} T cells into WT hosts which were subsequently immunized with HEL-OVA/adjuvant and their GCs (IgD^{low} CD95^{+} GL7^{+}) were analyzed on day 8. (C) Frequencies of Bcl2-tg SW$_{HEL}$ DZ cells with high and low BCR levels that carry *Ighv* region premature stop codons are shown (left). The phenotype of sequenced cells with stop codons is shown to right. (D) Summary of results from multiple mice and experiments. Numbers of cells sequenced are indicated. Mouse 1 DZ is gated using just CXCR4. (E) Frequencies of DZ and LZ cells with premature stop codons. Numbers are means from two independent experiments which are shown separately in Figure S5F. Data in (A) and (B) are pooled from 3 experiments with each point representing a single mouse. Analysis was performed using an unpaired two-tailed Student's t test, ***$p < 0.001$. See also Figure S5.

via a tight selection "bottleneck" (Bannard and Cyster, 2017). The introduction of somatic mutations is a mostly random process that is far more likely to cause detrimental changes to the immunoglobulin genes or proteins than it is to increase antigen binding. A major challenge for the GC response must therefore be to quickly screen out cells in which SHM has been harmful to limit their impact on the response as a whole. The presence of a relatively small number of newly minted high-affinity receptors on the surface of cells is probably sufficient for their positive selection even when old BCR complexes are also present, but screening out B cells bearing loss-of-function immunoglobulin mutations will require the exchange of pre-SHM receptors with the new variants.

In this study, we found that BCR replacement following SHM occurred mostly within a single DZ cycle, and as such is likely to be quite rapid process. Cells carrying stop codons were approximately 3-fold more abundant in DZs than in LZs and most stop codon^{+} LZ cells had high surface BCR expression. It is important to consider that some DZ cells might have entered that phase already carrying damaged receptor genes due to their still being BCR^{high} at the time of selection; however, such cells represented only ~1% of the LZ. Consequently, most of the DZ cells with damaged receptors (~80%) were newly generated and two thirds had decreased their surface BCR levels. Moreover, these calculations may underestimate the true extent of BCR turnover in the DZ because they do not take into account stop codon^{+} BCR^{low} cells that died before being counted. While we restricted our analysis to stop codons for feasibility reasons, we predict similar consequences for GC B cells acquiring other types of mutations that cause loss of functional BCR complexes. However, our results also indicate that the system is not foolproof because stop codon^{+} BCR^{high} LZ cells were rare but present and probably represent examples of post-SHM cells engaging in the selection process before replacing their antigen receptors.

Early studies on BCR turnover in follicular B cells predicted protein half-lives to be in the range of 20–80 hr (Andersson et al., 1974). However, similar turnover rates in GC B cells are unlikely to fit with the findings reported here. Faster turnover in the DZ provides opportunity for screening out cells with

detrimental mutations even before they return to the LZ and also means that once non-crippled cells reach there they are less likely to still carry ''old'' pre-SHM receptors that might otherwise cause inappropriate selection. Pioneering studies from the Rajewsky lab demonstrated that resting B cells depend upon tonic BCR derived signals for their survival, although in that situation BCR-less cells have half-lives of several days which is the equivalent to several LZ:DZ cycles (Kraus et al., 2004). This concept of receptor replacement in the DZ seems all the more important when considering the GC to be an antibody diversifier because the major challenge is perhaps to screen-out cells that have lost the ability to bind antigen rather than to preferentially pick the best clones.

The exact nature, kinetics, and mechanism of BCR replacement in the GC remain to be fully explored. Conceptually, only two possibilities exist for the events occurring after SHM; either old pre-mutation receptors are joined by the new somatic variants leading to a period of ''dual specificity,'' or pre-existing membrane receptor complexes must be rapidly degraded and replaced with new ones. The existence of small numbers of BCR^{high} cells carrying detrimental IgV region mutations in the DZ indicates that SHM occurs at times of receptor expression, however the greater abundance of stop codons within BCR^{low} populations suggests that replacement may then proceed quickly. The finding that some BCR^{low} DZ cells were capable of re-expressing their antigen receptors *ex vivo* raised the possibility that this subset may be a medley of cells; some engaged in a process of receptor replacement and others that carry damaging mutations that will cause them to soon die. Given the dependence of this subset upon AID, one hypothesis might be that SHM activity is sensed through DNA damage response pathways that subsequently initiate BCR degradation. However, this remains a purely speculative model and it is not clear whether BCR re-expression by BCR^{low} cells can or does occur *in vivo*. It is equally plausible that receptor replacement is a relatively rapid but continuous process and that the seemingly non-crippled BCR^{low} cells observed here carry mutations that destabilize or reduce, rather than completely disrupt, the membrane BCR complex.

Previous studies identified AID as being at its most active during G1 stages of cell cycle *in vitro* (Le and Maizels, 2015; Wang et al., 2017a) and our observation that BCR^{high} cells with stop codons were preferentially within G1 populations in GCs suggests that the same may hold true during SHM *in vivo*. Selection is initiated in the LZ while cells are in G1 and this drives their entry into S phase. As such, GC B cells should in most cases complete at least one division following their positive selection before undergoing further diversification; this might ensure that selected mutations are expanded in advance of further gene modification. Whether the fate of both daughter cells is the same in terms of subsequent AID activity, or whether instead one cell temporarily acts as a clonal amplifier, is not clear (Tas et al., 2016; Thaunat et al., 2012).

It has long been known that GCs are sites of extensive apoptotic cell death; cells failing to receive adequate T cell help during selection are thought to mostly die by neglect (Liu et al., 1989; Peperzak et al., 2012; Smith et al., 2000; Takahashi et al., 1999). While GC B cells with active caspase3 were found in both zones, our findings suggest that the majority of cell death occurs when in the DZ state and therefore not seemingly as a direct and immediate consequence of failing selection for cyclic re-entry. This conclusion is consistent with a recent study by *Mayer et al.* that was published while our work was in preparation (Mayer et al., 2017). The DZ is a site of rapid cell division and, while cells of that zone may undergo apoptosis at any point during cell cycle, cell death was most common at late G1. We describe this period as a fate junction because it corresponds to a specific phase at which DZ cells are destined either to undergo another cell division or to enter the LZ, or seemingly to die. The latter may be the default pathway when cues or pathways that drive the former processes are not intact.

Based upon observations that positioning GC B cells in the LZ is not enough to make them look or behave like cells of that zone, it was proposed that the DZ to LZ transition is controlled by a ''timer'' that is part of an intrinsic cellular program (Bannard et al., 2013). This model was supported by later reports that T cell derived signals set the DZ dwell time and that the transcriptional regulator FoxO1 contributes to driving that state (Chou et al., 2016; Dominguez-Sola et al., 2015; Gitlin et al., 2015; 2014; Sander et al., 2015). However, these new findings that GC B cells acquiring detrimental IgV region mutations fail to reach the LZ, and that cells with low BCR levels preferentially die at a period that corresponds to when other cells are decreasing their CXCR4 levels in order to move to the LZ, raises the possibility that BCR dependent cues might feed into controlling this transition. Inputs through the BCR may enable LZ transitioning in a more active manner than just through enhanced survival because expression of a *Bcl2*-transgene was not sufficient to allow cells with damaged BCRs to reach the LZ. One possibility is that timed inactivation of FoxO1 leads to de-repression of BCR signaling in late DZ cells, as was recently proposed (Mesin et al., 2016). It may be relevant therefore that temporal deletion of Syk in GC B cells was very recently demonstrated to lead to greatly diminished LZ compartments (Luo et al., 2018). The dynamics and nature of such signals, whether they be tonic or antigen driven, and whether they relate to transient changes in responsiveness at particular times, will be important questions for further investigation (Khalil et al., 2012; Kraus et al., 2004; Nowosad et al., 2016; Srinivasan et al., 2009).

We were surprised to discover during the course of this work that the DZs of Bcl2-tg mice contain aberrant populations of seemingly quiescent cells. This mouse line has been utilized in the study of GC responses for approximately a quarter of a century with a seminal finding being that such mice develop enlarged memory B cell compartments (Smith et al., 1994). It is therefore of interest that appreciable fractions of the quiescent DZ cells in these mice express recently described pre-memory markers. Whether such cells develop directly from the DZ after exhausting their proliferative program or whether they instead they come via the LZ was not clear from our BrdU chase experiments. Importantly, although aberrant DZ cells were not excluded from our analysis of stop codon frequencies in SW_{HEL} x Bcl2-tg GCs because these mice did not carry the Fucci2 allele, their likely presence would not change the expectation that higher frequencies of stop codons should be seen (but were not) in the LZs of Bcl2-tg mice, as compared to the wild-type setting, if enhancing survival were all that was needed to facilitate the DZ to LZ transition.

GCs are specialized sites of remarkable genetic diversification and clonal evolution that occurs with impressive pace and efficiency. In the arms battle against pathogens, antibody diversification in GCs provides the plasticity needed for potentially developing antibodies that can neutralize a practically infinite range of antigens. But for the best clones to be selected after SHM, non-useful reaction by-products must be quickly screened out. Our study suggests that GC B cells are uniquely programmed to deal with this challenge. DZ cells appear to quickly turnover, and potentially replace, their receptors following SHM and as such cells acquiring harmful mutations die off in advance of LZ entry. For cells making it back to the LZ, BCRs will mostly match genes by that time, and therefore the focus of selection may be on efficiently testing whether their somatically mutated antibodies remain capable of antigen recognition. The evolutionary advantage seems likely to be facilitating the kinds of diversification dependent antibody maturation pathways that are required to counter complex pathogens that will do all that they can to avoid recognition.

STAR★METHODS

Detailed methods are provided in the online version of this paper and include the following:

- KEY RESOURCES TABLE
- CONTACT FOR REAGENT AND RESOURCE SHARING
- EXPERIMENTAL MODEL DETAILS
  - Mice
- METHOD DETAILS
  - Immunisation, treatments and adoptive transfer
- MIXED BONE MARROW CHIMERAS
- FLOW CYTOMETRY AND SINGLE CELL SORTING
- IMMUNOGLOBULIN HEAVY CHAIN SEQUENCING
  - Sanger sequencing
  - Library preparation and analysis for Ighv NGS sequencing
- *IN VITRO* CULTURE
  - Single cell culture
  - Short term cultures
- ELISA
- QUANTIFICATION AND STATISTICAL ANALYSIS
  - Statistical analysis
  - Calculating frequencies of cells carrying stop codons from sanger sequencing
  - Estimates of expected premature stop codon frequency

SUPPLEMENTAL INFORMATION

Supplemental Information includes five figures and can be found with this article online at https://doi.org/10.1016/j.immuni.2018.08.025.

ACKNOWLEDGMENTS

We are grateful to C. Anzilloti, R. Brink, R. Cornall, T. Honjo, S. Jayaram, C. Rada, and S. Srinivas for kindly providing, or allowing use of, various mouse strains. We thank T. Fujimori and RIKEN BDR Laboratories for Animal Resource Development and Genetic Engineering (LARGE) for R26p-Fucci2 mice. We also thank G. Kelsoe and M. Kuraoka for generously allowing use of NB21 cells and for help with protocols, and J. Cyster, J. Muppidi, and A. Reboldi for critical reading of the manuscript. N. Ashley (WIMM single cell core) provided technical advice and T. Rostron (WIMM sequencing core) provided sequencing services. Cell sorts were performed in the WIMM FACS facility with expert assistance from C. Waugh and S. Clarke. This work was funded by a Sir Henry Dale Fellowship from The Wellcome Trust/Royal Society (O.B., 105654/Z/14/Z) and by the Medical Research Council (UK) through core funding to the MRC Human Immunology Unit and the MRC WIMM (MC_UU_12010/3).

AUTHOR CONTRIBUTIONS

I.S. performed most of the experiments with help from O.B. and B.P. D.R. performed some BrdU chase experiments and assisted with indexed data processing. S.J.M. helped analyze Illumina sequencing data. I.S. and O.B. designed and analyzed all experiments and wrote the manuscript. O.B. devised and supervised the project.

DECLARATION OF INTERESTS

The authors declare no competing interests.

Received: December 24, 2017
Revised: April 23, 2018
Accepted: August 28, 2018
Published: September 18, 2018

REFERENCES

Abe, T., Sakaue-Sawano, A., Kiyonari, H., Shioi, G., Inoue, K., Horiuchi, T., Nakao, K., Miyawaki, A., Aizawa, S., and Fujimori, T. (2013). Visualization of cell cycle in mouse embryos with Fucci2 reporter directed by Rosa26 promoter. Development *140*, 237–246.

Allen, C.D.C., Ansel, K.M., Low, C., Lesley, R., Tamamura, H., Fujii, N., and Cyster, J.G. (2004). Germinal center dark and light zone organization is mediated by CXCR4 and CXCR5. Nat. Immunol. *5*, 943–952.

Allen, C.D.C., Okada, T., and Cyster, J.G. (2007a). Germinal-center organization and cellular dynamics. Immunity *27*, 190–202.

Allen, C.D.C., Okada, T., Tang, H.L., and Cyster, J.G. (2007b). Imaging of germinal center selection events during affinity maturation. Science *315*, 528–531.

Andersson, J., Lafleur, L., and Melchers. (1974). IgM in bone marrow-derived lymphocytes. Synthesis, surface deposition, turnover and carbohydrate composition in unstimulated mouse B cells. Eur. J. Immunol. *4*, 170–180.

Bannard, O., and Cyster, J.G. (2017). Germinal centers: programmed for affinity maturation and antibody diversification. Curr. Opin. Immunol. *45*, 21–30.

Bannard, O., Horton, R.M., Allen, C.D.C., An, J., Nagasawa, T., and Cyster, J.G. (2013). Germinal center centroblasts transition to a centrocyte phenotype according to a timed program and depend on the dark zone for effective selection. Immunity *39*, 912–924.

Bannard, O., McGowan, S.J., Ersching, J., Ishido, S., Victora, G.D., Shin, J.-S., and Cyster, J.G. (2016). Ubiquitin-mediated fluctuations in MHC class II facilitate efficient germinal center B cell responses. J. Exp. Med. *213*, 993–1009.

Barnden, M.J., Allison, J., Heath, W.R., and Carbone, F.R. (1998). Defective TCR expression in transgenic mice constructed using cDNA-based alpha- and beta-chain genes under the control of heterologous regulatory elements. Immunol. Cell Biol. *76*, 34–40.

Batista, F.D., and Neuberger, M.S. (2000). B cells extract and present immobilized antigen: implications for affinity discrimination. EMBO J. *19*, 513–520.

Berek, C., Berger, A., and Apel, M. (1991). Maturation of the immune response in germinal centers. Cell *67*, 1121–1129.

Chou, C., Verbaro, D.J., Tonc, E., Holmgren, M., Cella, M., Colonna, M., Bhattacharya, D., and Egawa, T. (2016). The Transcription Factor AP4 Mediates Resolution of Chronic Viral Infection through Amplification of Germinal Center B Cell Responses. Immunity *45*, 570–582.

Dominguez-Sola, D., Kung, J., Holmes, A.B., Wells, V.A., Mo, T., Basso, K., and Dalla-Favera, R. (2015). The FOXO1 Transcription Factor Instructs the Germinal Center Dark Zone Program. Immunity *43*, 1064–1074.

Gitlin, A.D., Shulman, Z., and Nussenzweig, M.C. (2014). Clonal selection in the germinal centre by regulated proliferation and hypermutation. Nature *509*, 637–640.

Gitlin, A.D., Mayer, C.T., Oliveira, T.Y., Shulman, Z., Jones, M.J.K., Koren, A., and Nussenzweig, M.C. (2015). HUMORAL IMMUNITY. T cell help controls the speed of the cell cycle in germinal center B cells. Science *349*, 643–646.

Goodnow, C.C., Crosbie, J., Adelstein, S., Lavoie, T.B., Smith-Gill, S.J., Brink, R.A., Pritchard-Briscoe, H., Wotherspoon, J.S., Loblay, R.H., Raphael, K., et al. (1988). Altered immunoglobulin expression and functional silencing of self-reactive B lymphocytes in transgenic mice. Nature *334*, 676–682.

Hahne, F., LeMeur, N., Brinkman, R.R., Ellis, B., Haaland, P., Sarkar, D., Spidlen, J., Strain, E., and Gentleman, R. (2009). flowCore: a Bioconductor package for high throughput flow cytometry. BMC Bioinformatics *10*, 106.

Jacob, J., Kelsoe, G., Rajewsky, K., and Weiss, U. (1991). Intraclonal generation of antibody mutants in germinal centres. Nature *354*, 389–392.

Kelsoe, G., and Haynes, B.F. (2017). Host controls of HIV broadly neutralizing antibody development. Immunol. Rev. *275*, 79–88.

Khalil, A.M., Cambier, J.C., and Shlomchik, M.J. (2012). B cell receptor signal transduction in the GC is short-circuited by high phosphatase activity. Science *336*, 1178–1181.

Kraus, M., Alimzhanov, M.B., Rajewsky, N., and Rajewsky, K. (2004). Survival of resting mature B lymphocytes depends on BCR signaling via the Igalpha/beta heterodimer. Cell *117*, 787–800.

Kuraoka, M., Schmidt, A.G., Nojima, T., Feng, F., Watanabe, A., Kitamura, D., Harrison, S.C., Kepler, T.B., and Kelsoe, G. (2016). Complex Antigens Drive Permissive Clonal Selection in Germinal Centers. Immunity *44*, 542–552.

Laidlaw, B.J., Schmidt, T.H., Green, J.A., Allen, C.D.C., Okada, T., and Cyster, J.G. (2017). The Eph-related tyrosine kinase ligand Ephrin-B1 marks germinal center and memory precursor B cells. J. Exp. Med. *214*, 639–649.

Le, Q., and Maizels, N. (2015). Cell Cycle Regulates Nuclear Stability of AID and Determines the Cellular Response to AID. PLoS Genet. *11*, e1005411.

Lingwood, D., McTamney, P.M., Yassine, H.M., Whittle, J.R.R., Guo, X., Boyington, J.C., Wei, C.-J., and Nabel, G.J. (2012). Structural and genetic basis for development of broadly neutralizing influenza antibodies. Nature *489*, 566–570.

Liu, Y.J., Joshua, D.E., Williams, G.T., Smith, C.A., Gordon, J., and MacLennan, I.C. (1989). Mechanism of antigen-driven selection in germinal centres. Nature *342*, 929–931.

Luo, W., Weisel, F., and Shlomchik, M.J. (2018). B Cell Receptor and CD40 Signaling Are Rewired for Synergistic Induction of the c-Myc Transcription Factor in Germinal Center B Cells. Immunity *48*, 313–326.e5.

Masella, A.P., Bartram, A.K., Truszkowski, J.M., Brown, D.G., and Neufeld, J.D. (2012). PANDAseq: paired-end assembler for illumina sequences. BMC Bioinformatics *13*, 31.

Mayer, C.T., Gazumyan, A., Kara, E.E., Gitlin, A.D., Golijanin, J., Viant, C., Pai, J., Oliveira, T.Y., Wang, Q., Escolano, A., et al. (2017). The microanatomic segregation of selection by apoptosis in the germinal center. Science *358*, eaao2602.

McKean, D., Huppi, K., Bell, M., Staudt, L., Gerhard, W., and Weigert, M. (1984). Generation of antibody diversity in the immune response of BALB/c mice to influenza virus hemagglutinin. Proc. Natl. Acad. Sci. USA *81*, 3180–3184.

Mesin, L., Ersching, J., and Victora, G.D. (2016). Germinal Center B Cell Dynamics. Immunity *45*, 471–482.

Muramatsu, M., Kinoshita, K., Fagarasan, S., Yamada, S., Shinkai, Y., and Honjo, T. (2000). Class switch recombination and hypermutation require activation-induced cytidine deaminase (AID), a potential RNA editing enzyme. Cell *102*, 553–563.

Nowosad, C.R., Spillane, K.M., and Tolar, P. (2016). Germinal center B cells recognize antigen through a specialized immune synapse architecture. Nat. Immunol. *17*, 870–877.

Pappas, L., Foglierini, M., Piccoli, L., Kallewaard, N.L., Turrini, F., Silacci, C., Fernandez-Rodriguez, B., Agatic, G., Giacchetto-Sasselli, I., Pellicciotta, G., et al. (2014). Rapid development of broadly influenza neutralizing antibodies through redundant mutations. Nature *516*, 418–422.

Peperzak, V., Vikstrom, I.B., and Tarlinton, D.M. (2012). Through a glass less darkly: apoptosis and the germinal center response to antigen. Immunol. Rev. *247*, 93–106.

Phan, T.G., Amesbury, M., Gardam, S., Crosbie, J., Hasbold, J., Hodgkin, P.D., Basten, A., and Brink, R. (2003). B cell receptor-independent stimuli trigger immunoglobulin (Ig) class switch recombination and production of IgG autoantibodies by anergic self-reactive B cells. J. Exp. Med. *197*, 845–860.

Purtha, W.E., Tedder, T.F., Johnson, S., Bhattacharya, D., and Diamond, M.S. (2011). Memory B cells, but not long-lived plasma cells, possess antigen specificities for viral escape mutants. J. Exp. Med. *208*, 2599–2606.

Rice, P., Longden, I., and Bleasby, A. (2000). EMBOSS: the European Molecular Biology Open Software Suite. Trends Genet. *16*, 276–277.

Sander, S., Chu, V.T., Yasuda, T., Franklin, A., Graf, R., Calado, D.P., Li, S., Imami, K., Selbach, M., Di Virgilio, M., et al. (2015). PI3 Kinase and FOXO1 Transcription Factor Activity Differentially Control B Cells in the Germinal Center Light and Dark Zones. Immunity *43*, 1075–1086.

Smith, K.G., Weiss, U., Rajewsky, K., Nossal, G.J., and Tarlinton, D.M. (1994). Bcl-2 increases memory B cell recruitment but does not perturb selection in germinal centers. Immunity *1*, 803–813.

Smith, K.G., Light, A., O'Reilly, L.A., Ang, S.M., Strasser, A., and Tarlinton, D. (2000). bcl-2 transgene expression inhibits apoptosis in the germinal center and reveals differences in the selection of memory B cells and bone marrow antibody-forming cells. J. Exp. Med. *191*, 475–484.

Srinivasan, L., Sasaki, Y., Calado, D.P., Zhang, B., Paik, J.H., DePinho, R.A., Kutok, J.L., Kearney, J.F., Otipoby, K.L., and Rajewsky, K. (2009). PI3 kinase signals BCR-dependent mature B cell survival. Cell *139*, 573–586.

Strasser, A., Whittingham, S., Vaux, D.L., Bath, M.L., Adams, J.M., Cory, S., and Harris, A.W. (1991). Enforced BCL2 expression in B-lymphoid cells prolongs antibody responses and elicits autoimmune disease. Proc. Natl. Acad. Sci. USA *88*, 8661–8665.

Suan, D., Kräutler, N.J., Maag, J.L.V., Butt, D., Bourne, K., Hermes, J.R., Avery, D.T., Young, C., Statham, A., Elliott, M., et al. (2017). CCR6 Defines Memory B Cell Precursors in Mouse and Human Germinal Centers, Revealing Light-Zone Location and Predominant Low Antigen Affinity. Immunity *47*, 1142–1153.e4.

Takahashi, Y., Cerasoli, D.M., Dal Porto, J.M., Shimoda, M., Freund, R., Fang, W., Telander, D.G., Malvey, E.N., Mueller, D.L., Behrens, T.W., and Kelsoe, G. (1999). Relaxed negative selection in germinal centers and impaired affinity maturation in bcl-xL transgenic mice. J. Exp. Med. *190*, 399–410.

Tas, J.M.J., Mesin, L., Pasqual, G., Targ, S., Jacobsen, J.T., Mano, Y.M., Chen, C.S., Weill, J.-C., Reynaud, C.-A., Browne, E.P., et al. (2016). Visualizing antibody affinity maturation in germinal centers. Science *351*, 1048–1054.

Thaunat, O., Granja, A.G., Barral, P., Filby, A., Montaner, B., Collinson, L., Martinez-Martin, N., Harwood, N.E., Bruckbauer, A., and Batista, F.D. (2012). Asymmetric segregation of polarized antigen on B cell division shapes presentation capacity. Science *335*, 475–479.

Victora, G.D., and Nussenzweig, M.C. (2012). Germinal centers. Annu. Rev. Immunol. *30*, 429–457.

Victora, G.D., Schwickert, T.A., Fooksman, D.R., Kamphorst, A.O., Meyer-Hermann, M., Dustin, M.L., and Nussenzweig, M.C. (2010). Germinal center dynamics revealed by multiphoton microscopy with a photoactivatable fluorescent reporter. Cell *143*, 592–605.

Victora, G.D., Dominguez-Sola, D., Holmes, A.B., Deroubaix, S., Dalla-Favera, R., and Nussenzweig, M.C. (2012). Identification of human germinal center light and dark zone cells and their relationship to human B-cell lymphomas. Blood *120*, 2240–2248.

Wang, Q., Kieffer-Kwon, K.-R., Oliveira, T.Y., Mayer, C.T., Yao, K., Pai, J., Cao, Z., Dose, M., Casellas, R., Jankovic, M., et al. (2017a). The cell cycle restricts activation-induced cytidine deaminase activity to early G1. J. Exp. Med. *214*, 49–58.

Wang, Y., Shi, J., Yan, J., Xiao, Z., Hou, X., Lu, P., Hou, S., Mao, T., Liu, W., Ma, Y., et al. (2017b). Germinal-center development of memory B cells driven by IL-9 from follicular helper T cells. Nat. Immunol. *18*, 921–930.

West, A.P., Jr., Scharf, L., Scheid, J.F., Klein, F., Bjorkman, P.J., and Nussenzweig, M.C. (2014). Structural insights on the role of antibodies in HIV-1 vaccine and therapy. Cell *156*, 633–648.

Yeap, L.-S., Hwang, J.K., Du, Z., Meyers, R.M., Meng, F.-L., Jakubauskaitė, A., Liu, M., Mani, V., Neuberg, D., Kepler, T.B., Wang, J.H., and Alt, F.W. (2015). Sequence-Intrinsic Mechanisms that Target AID Mutational Outcomes on Antibody Genes. Cell *163* (5), 1124–1137, http://doi.org/10.1016/j.cell.2015.10.042.

STAR★METHODS

KEY RESOURCES TABLE

REAGENT or RESOURCE	SOURCE	IDENTIFIER
Antibodies		
APC Cy7 B220, clone Ra3 6b2	Biolegend	Cat# 103224; RRID:AB_313006
BV785 B220, clone Ra3 6b2	Biolegend	Cat# 103245; RRID: AB_11218795
FITC B220, clone Ra3 6b2	Biolegend	Cat# 103206; RRID: AB_312990
FITC CD19, clone 6d5	Biolegend	Cat# 115506; RRID: AB_313640
BV421 CD138, clone 2812	Biolegend	Cat# 356516; RRID: AB_2562659
PerCP Cy5.5 IgD, clone 11-26c-2a	Biolegend	Cat# 405710; RRID: AB_1575115
BUV 395 IgD, clone 11-26c-2a	BD bioscience	Cat# 565988; RRID:AB_2737433
PE Cy7 CD95, clone Jo2	BD bioscience	Cat# 557653; RRID:AB_396768
APC Cy7 CD38, clone 90	Biolegend	Cat# 102728; RRID:AB_2616967
PB T/B cell activation marker, clone GL7	Biolegend	Cat# 144614; RRID:AB_2563291
PE CD79b, clone HM79 12	Biolegend	Cat# 132804; RRID:AB_1575061
FITC CD79b, clone HM79 12	Biolegend	Cat# 132806; RRID:AB_2244530
PerCP Cy5.5 CD79b, clone HM79 12	Biolegend	Cat# 132810; RRID:AB_2632917
Purified CD79b, clone HM79 12	Biolegend	Cat# 132802; RRID:AB_1575063
PE IgG1, clone Rmg1-1	Biolegend	Cat# 406608; RRID:AB_10551439
PE IgMa, clone MA-69	Biolegend	Cat# 408608; RRID:AB_940545
FITC IgMa, clone MA-69	Biolegend	Cat# 408606; RRID:AB_940541
Biotin CXCR4 (CD184), clone 2B11	Biolegend	Cat# 146516; RRID:AB_2650787
AF647 CD83, clone Michel-19	Biolegend	Cat# 121514; RRID:AB_2074746
PE Cy7 CD83, clone Michel-19	Biolegend	Cat# 121518; RRID:AB_2566123
PE Ly5.2 (CD45.1), clone A20	Biolegend	Cat# 110708; RRID:AB_313496
FITC Ly5.2 (CD45.1), clone A20	Biolegend	Cat# 110706; RRID:AB_313494
PerCP Cy5.5 Ly5.2 (CD45.1), clone A20	Biolegend	Cat#110728; RRID:AB_893348
FITC Ly5.1 (CD45.2), clone 104	Biolegend	Cat#109806; RRID:AB_313442
PerCP Cy5.55 Ly5.1 (CD45.2), clone 104	Biolegend	Cat#109828; RRID:AB_893352
BV785 Ly5.1 (CD45.2), clone 104	Biolegend	Cat# 109839;RRID:AB_2562604
APC Cy7 Igκ, clone RMK-45	Biolegend	Cat# 409504; RRID:AB_2563578
Active Caspase 3, clone C92-605	BD pharmingen	Cat# 559565; RRID:AB_397274
BCL6, clone K112-91	BD biosciences	Cat#561525; RRID:AB_10898007
AF647 CCR6, clone 29-2L17	Biolegend	Cat# 129808; RRID:AB_1227497
APC Cy7 CD62L, clone MEL-14	Biolegend	Cat# 104428; RRID:AB_830799
Biotin goat anti- Ephrin B1	R&D Systems	Cat# BAF473; RRID:AB_2293418
PB BRDU antibody, clone MoBU-1	Invitrogen	Cat# B35129; RRID:AB_2536433
Purified Igk, polyclonal	Southern biotech	Cat# 1050-01; RRID:AB_2737431
HRP anti-mouse IgG, polyclonal	Southern biotech	Cat# 1033-05; RRID:AB_2737432
Experimental models: Organisms/strains		
SW_{HEL} mice	Phan et al., 2003	N/A
MD4 transgenic mice	Goodnow et al., 1988	N/A
OT-II transgenic mice	Barnden et al., 1998	N/A
R26p-Fucci2 mice	Abe et al., 2013, http://www.clst.riken.jp/arg/reporter_mice.html	Riken accession # CDB0203T
Bcl-2 transgenic mice	Strasser et al., 1991	N/A
Aicda$^{-/-}$ mice	Muramatsu et al., 2000	N/A

(*Continued on next page*)

Continued

REAGENT or RESOURCE	SOURCE	IDENTIFIER
C57BL/6 mice	Oxford University core breeding facility	N/A
B6SJLCD45.1 mice	Oxford University core breeding facility	N/A
Experimental models: cell lines		
NB-21.2D9 feeder cells	Kuraoka, M. et al. Complex Antigens Drive Permissive Clonal Selection in Germinal Centers. *Immunity* **44,** 542–552 (2016).	N/A
Oligonucleotides		
1st Step SW_{HEL} forward (GTTGTAGCCTAAAAGATGATGGTG)	Integrated DNA Technologies	N/A
1st Step SW_{HEL} reverse (GATAATCTGTCCTAAAGGCTCTGAG)	Integrated DNA Technologies	N/A
2nd Step SW_{HEL} forward (TTGTAGCCTAAAAGATGATGGTGTTAAGTC)	Integrated DNA Technologies	N/A
2nd Step SW_{HEL} forward (CAACTTCTCTCAGCCGGCTC)	Integrated DNA Technologies	N/A
Chemical, peptides, and recombinant proteins		
Lysosyme from chicken egg white (Hen Egg Lysozyme, HEL)	Sigma Aldrich	L6876-5g
Albumin from chicken egg white	Sigma Aldrich	A5503-5g
Sigma Adjuvant system	Sigma Aldrich	S6322-1VL
Baytril	Bayer	N/A
Fisher BioReagents Proteinase K	Fisher Scientific	Cat# 10172903
Recombinant murine IL-4	Peprotech	Cat# 214-14
Q5 High Fidelity DNA polymerase	New England Biolabs	Cat# M0491L
AFIIII	New England Biolabs	Cat# R0541S
NsiI-HF	New England Biolabs	Cat# R3127S
Critical commercial assays		
Streptavidin Qdot 605	Thermo fisher scientific	Cat# A63881
AF467, Alexa Fluor 647 Protein Labeling Kit	Thermo fisher scientific	Cat# A20173
TMB Substrate Set	Biolegend	Cat# 421101
Stop solution for TMB substrate	Biolegend	Cat# 423001
eBioscience Foxp3 / Transcription Factor Staining Buffer Set	Invitrogen	Cat# 00-5523-00
Fixation/Permeabilization Solution Kit	BD Biosciences	Cat# 554714
Permeabilization Buffer Plus	BD Biosciences	Cat# 561651
NEBNext® Ultra II DNA Library Prep Kit for Illumina®	New Enlgand Biolabs	Cat# E7645S
NEBNext® Multiplex Oligos for Illumina® (Dual Index Primers Set 1)	New England Biolabs	Cat# E7600S
USER® Enzyme	New England Biolabs	Cat# M5505S
Adaptor for Illumina	New England Biolabs	Cat# E7337G
MiSeq Reagent Kit v2 (500-cycles)	Illumina	Cat# MS-102-2003
5-Bromo-2′-deoxyuridine	Sigma Aldrich	Cat# B5002
Qubit dsDNA HS Assay Kit	Invitrogen	Cat# Q32854
Software and algorithms		
FlowJo 10.1r5 (Treestar inc)	https://www.flowjo.com/	RRID:SCR_008520
Graphpad prism version 7	https://www.graphpad.com/scientific-software/prism/	RRID:SCR_002798
IgBlast tool (NCBI)	https://www.ncbi.nlm.nih.gov/igblast/	RRID:SCR_002873

(*Continued on next page*)

Continued

REAGENT or RESOURCE	SOURCE	IDENTIFIER
flowCore, R package version 1.44.0	http://bioconductor.org/packages/release/bioc/html/flowCore.html	RRID:SCR_002205
Adobe Illustrator CC 2018	https://www.adobe.com/uk/products/illustrator.html	RRID:SCR_014198
PANDAseq	https://doi.org/10.1186/1471-2105-13-31	RRID:SCR_002705
Needle	https://www.ebi.ac.uk/Tools/psa/emboss_needle/	N/A
Custom perl scripts (find_stop_codons.pl)	http://sara.molbiol.ox.ac.uk/public/obannard/scripts/find_stop_codons_pl.txt	N/A
Custom perl scripts (make_summary.pl).	http://sara.molbiol.ox.ac.uk/public/obannard/scripts/make_summary_pl.txt	N/A
Miscellaneous		
Agencourt AMPure XP PCR purification beads	Beckman coulter	Cat# A63881
Tween20	Sigma Aldrich	Cat# 9005-64-5
Triton-x	Sigma Aldrich	Cat# 9002-93-1
Fetal Bovine Serum, qualified, heat inactivated	GIBCO	Cat# 10500 056
Fetal bovine serum	GIBCO	Cat# 10500064
HEPES	GIBCO	Cat# 15630-054
Glutamine	Thermo fisher scientific	Cat# 21051040
Sodium Pyruvate	GIBCO	Cat# 11360-039
Penicillin and Streptomycin	Thermo fisher scientific	Cat# 15140122
MEM Non-essential amino acids solution (x100)	Thermo fisher scientific	Cat# 11140050
Normal mouse serum	Thermo fisher scientific	Cat# 24-5544-94
tRNA	Thermo fisher scientific	Cat# AM7119
EDTA	Invitrogen	55687
Sheep red blood cells	Scientific laboratory supplies	Cat# SR0051B

CONTACT FOR REAGENT AND RESOURCE SHARING

Further information and requests for resources and reagents should be directed to and will be fulfilled by the Lead Contact, Oliver Bannard (oliver.bannard@ndm.ox.ac.uk). The mouse lines obtained from other laboratories are described above and may require a Material Transfer Agreement (MTA) with the providing scientists.

EXPERIMENTAL MODEL DETAILS

Mice

SW_{HEL} (Phan et al., 2003), MD4 (Goodnow et al., 1988)), OT-II (Barnden et al., 1998), R26p-Fucci2 (Abe et al., 2013), Emu-Bcl-2tg (Strasser et al., 1991) and *Aicda*$^{-/-}$ (Muramatsu et al., 2000) mice have been described previously. C57BL/6 and B6SJLCD45.1 mice were purchased from the University of Oxford core breeding facility. Animals were housed in specific pathogen–free enclosures at the University of Oxford Biomedical Sciences facility. All experiments were approved by a project license granted by the UK Home Office and were also approved by the Institutional Animal Ethics Committee Review Board at the University of Oxford. All mice were over 6 weeks of age and a mixture of male and female mice were used.

METHOD DETAILS

Immunisation, treatments and adoptive transfer

For HEL-specific GC responses, $5x10^4$ to $1x10^5$ MD4, SW_{HEL} or SW_{HEL}xFucci2 cells were co-transferred with an equal number of OT-II cells into C57BL/6 or B6SJLCD45.1 hosts by intravenous injection. Mice were immunized one to three days later with 50ug of HEL conjugated to ovalbumin (HEL-OVA) in 100uL of Sigma adjuvant system (Ribi)(200ul total volume) as previously described (Allen et al., 2007b). For polyclonal responses, mice were immunized with $\sim 2 \times 10^8$ defribinated SRBCs (Fisher Scientific) by i.p. injection. For *in vivo* BrdU labeling experiments, mice received single i.p. injections of 2mg BrdU in saline.

MIXED BONE MARROW CHIMERAS

To generate mixed bone marrow chimeric mice, B6SJLCD45.1 host mice were lethally irradiated at 4.5 Gy for 300 s, followed by a 3 hour rest, and a subsequent 4.5 Gy dose for 300 s. Mice were injected i.v. with a 2:1 mixture CD45.1 WT and CD45.2 *Aicda*$^{-/-}$ or *Aicda*$^{+/+}$ bone marrow. Recipient mice received drinking water containing antibiotics (0.16mg/mL Enrofloxacin (Baytril), Bayer Coporation). Mice were rested for > 8 weeks before use.

FLOW CYTOMETRY AND SINGLE CELL SORTING

Single cell suspensions were prepared and stained for with the relevant antibodies. BRDU staining was performed following the fixation/permeabilization reagents from the BD BrdU flow kit according to manufacturer's instructions but using the Mobu-1 antibody clone (Thermo Fisher Scientific). Intranuclear BCL6 staining was performed on cells permeabilised using the eBioscience Foxp3 / Transcription Factor Staining Buffer Set (Invitrogen). Active caspase 3 staining was performed on cells permeabilised using the Fixation/Permeabilization Solution Kit (BD bioscience). All permeablization steps were performed with an overnight incubation at 4°C. Samples were analyzed using BD LSR Fortessa, LSR Fortessa x20, LSR Fortessa x50, LSR II, or Attune NxT machines. Analysis was performed with Flowjo (Treestar Inc.). Single cell index sorting for sequencing and cell culture experiments were performed using the BD FACs Aria II, FACs Aria II SORP, or Fusion II machines. FACs sorts were performed using 85uM or 100uM (when culturing) nozzles.

IMMUNOGLOBULIN HEAVY CHAIN SEQUENCING

Sanger sequencing

Single cells were sorted into 3.8uL of 0.2% Triton-x (Sigma Aldrich) in water. Proteinase K treatment was performed in 1X Q5 reaction buffer, with 1mg/mL tRNA; 1mg/mL proteinase K, and 0.05mM EDTA in a total volume of 10uL by heating to 56°C for 40 minutes followed by 95°C for 8 minutes. PCR was performed with Q5 High Fidelity DNA polymerase (New England Biolabs), and dNTPs (Thermo Fisher Scientific). Primary PCR used primers "1st Step SW_{HEL} forward" and "1st Step SW_{HEL} reverse." Conditions were 98°C for 3 minutes; then 98°C for 15 s, 62°C for 1 minute, and 72°C for 1 minute, for 35 cycles; and finally 3 minutes at 72°C. A 1:50 dilution of the initial product was used as template for the second step PCR. The 2nd step PCR was performed using primers "2nd Step SW_{HEL} forward" and "2nd Step SW_{HEL} reverse." Conditions were 98°C for 3 minutes; then 35 cycles of 98°C for 15 s, 65°C for 30 s, then 72°C for 1 minute; and finally 3 minutes at 72°C. The PCR had an approximately 80% efficiency. PCR was checked by running 1uL of product on a 96 well agarose gel. PCR product purification was performed using Agencourt AMPure XP PCR purification beads (Beckman coulter). The concentration of DNA was measured by nanodrop. Sanger sequencing was performed by the Weatherall Institute of Molecular Medicine's sequencing unit.

Sanger sequences were converted to FASTA files and analyzed using NCBI IgBlast tool to search for premature stop codons. Sequencing data was combined with Indexed FACS data, and converted to an FCS file using, R package version 1.44.0. (Hahne et al., 2009). Original Fcs files and sequence Fcs files were superimposed using Adobe Illustrator

Library preparation and analysis for Ighv NGS sequencing

Bulk populations were sorted in 7.6uL of 0.2% Triton-x (Sigma Aldrich) in water. The PCR was performed as described for single cells with the exception that the initial PCR involved only 20 cycles. DNA was purified using Agencourt AMPure XP PCR purification beads (Beckman coulter), and the DNA products were sequentially cut with NsiI-HF and AFIIIII to produce a ~435bp product. The DNA was subsequently purified, end prep and adaptor ligation was performed using NEBNext® Ultra II DNA Library Prep Kit for Illumina® (New Enlgand Biolabs, E7645S) according to the manufacturer's instructions and NEBNext® Multiplex Oligos for Illumina® (New England Biolabs, E7600S) were added according to the manufacturers instructions. The DNA was again purified, quantified using Qubit dsDNA HS Assay Kit (Invitrogen, Q32854). The DNA was sequenced on an Illumina MiSeq using MiSeq Reagent Kit v2 (500-cycles) (Illumina, MS-102-2003)

PANDAseq was used to assemble paired-end FASTQ reads resulting from sequence analysis (Masella et al., 2012). The assembled sequences were compared to the known SW_{HEL} *Ighv* genomic template sequence by pairwise sequence alignment using needle (Rice et al., 2000). Following translation of the DNA sequence, STOP codons were identified in the relevant forward frame. Custom perl scripts were used to automate an analysis pipeline (find_stop_codons.pl) and summarize the subsequent data generated (make_summary.pl).

The number of reads of a given unique sample should be proportional to the number of times the read is identified in a sample. If 100 cells were sorted, then one would expect a sequence which occurred in only one cell, to be present in 1% of the reads; if 500 cells were sorted, one would expect a sequence which occurred in only one cells to be present in 0.2% of reads. Therefore, we selected only reads which occurred in more than (100/n)% of reads for that given sample. With this threshold, an average of 49.7% of reads appeared to have a somatic hypermutation of any kind in *Aicda*$^{+/+}$ samples, and an average of 1.3% of reads from *Aicda*$^{-/-}$ samples appeared to have a SHM of any kind. The frequency of reads carrying a premature stop codon is the combined number of reads carrying a premature stop codon from sequences passing this threshold, divided by the total number of reads passing threshold, multiplied by 100 to give a percentage value.

IN VITRO CULTURE

Single cell culture

Single GC B cells were FACs sorted and expanded for 8 days using the NB21 feeder line as previously described, with minor adjustments (Kuraoka et al., 2016). On day −1, NB21.2D9 cells were seeded into 96-well plates at 1,000 cells/well in 100uL of B cell media (RPMI-1640 (Invitrogen), (Thermo Scientific), 10% FBS (GIBCO, Cat #10500 056) 5.5x10^{-5}M 2-mercaptoethanol (Sigma Aldrich), 10 mM HEPES, (GIBCO) 1mM sodium pyruvate, (GIBCO) 100 units/mL penicillin, (Thermo Fisher) 100 mg/mL streptomycin, and MEM nonessential amino acid (Thermo fisher)). On day 0, recombinant mouse IL-4 (Peprotech; 2 ng/mL final concentration) was added in a further 100ul media which single cells were subsequently sorted into. On day 3, 100uL of media was replaced with 150uL of fresh media (without IL-4). 150uL media was subsequently discarded and replaced on days 5, 6 and 7. One row on each plate was left blank as controls for cross well contamination. Cell growth was confirmed by flow cytometry, detecting either endogenous mCherry florescence (Fucci2) or after staining with CD19/CD138 antibodies. Thresholds for detecting lymphocytes versus feeder cells were established using NB21.2D9 cell alone wells.

Short term cultures

For 8 hour B cell cultures, 1000 cells were sorted into 200uL of B cell medium without feeder cells. For 48 hour cultures, sorted B cells were cultured in the presence of NB21 feeder cells as described above. 1000 cells were sorted either straight onto NB21 feeder line cells or into media and then transferred into wells containing the cells, and cultured in the presence of IL-4. For both 8 hour and 48 hour cultures, three technical replicates were taken for each sample.

ELISA

The capacity of cultured B cells to secrete antibodies was assessed by ELISA. Plates were coated O/N at 4°C with anti-mouse Igκ (2ug/ml, Southern Biotech, 1050-01), washed 3x in PBS/0.05% Tween20, blocked for 1hr at RT with 0.5% BSA/PBS and washed again as before. Samples were diluted in PBS/0.05% Tween20 before incubation for 2hrs at RT or O/N at 4°C. Plates are washed as before, and the secondary HRP anti-mouse IgG antibody (Southern Biotech, 1033-05) was added at 1:2000 in 1x PBS/0.05% Tween20/0.5% BSA. Cells were washed as before, and plates were developed with TMB (Biolegend). Reactions were stopped with 50ul of Biolegend stop reagent and read on CLARIOstar at 450nm against a mouse IgG standard curve. Samples were scored as positive if they were > 4 standard deviations above the mean of the negative controls.

QUANTIFICATION AND STATISTICAL ANALYSIS

Statistical analysis

Statistical parameters including number of mice, number of replicates, and the nature of error bars are described in figure legends. Center bars always indicate means. Statistics were calculated using Prism 7 software (Graphpad). Two-tailed unpaired t tests were used. ***$p < 0.001$.

Calculating frequencies of cells carrying stop codons from sanger sequencing

During sorting for *Ighv* sequence analysis, minority populations and/or populations of interest were often enriched, for example BCR^{low} or specific cell cycle stage cells that are a small fraction of total LZ/DZ subsets. Consequently, in calculating frequencies of stop codons in parental (e.g., DZ) populations, frequencies were normalized to the abundance of their parental populations.

Frequencies of stop codons in BCR^{high} and BCR^{low} LZs and DZs were calculated as follows (example from BCR^{low} DZ):

$$
\begin{aligned}
&\textit{frequency of stop codons in BCR}^{low}\textit{ DZ}\\
&= \textit{frequency of stop codons in mCherry}^{low}\textit{ Venus}^{low}\textit{ BCR}^{low}\textit{DZ} \times \frac{\%\textit{mCherry}^{low}\textit{ Venus}^{low}\textit{ in BCR}^{low}\textit{ DZ}}{100}\\
&+ \textit{frequency of stop codons in mCherry}^{high}\textit{ Venus}^{low}\textit{ BCR}^{low}\textit{DZ} \times \frac{\%\textit{mCherry}^{low}\textit{ Venus}^{high}\textit{ in BCR}^{low}\textit{ DZ}}{100}\\
&+ \textit{frequency of stop codons in mCherry}^{low}\textit{ Venus}^{high}\textit{ BCR}^{low}\textit{DZ} \times \frac{\%\textit{mCherry}^{low}\textit{ Venus}^{high}\textit{ in BCR}^{low}\textit{ DZ}}{100}\\
&+ \textit{frequency of stop codons in mCherry}^{high}\textit{ Venus}^{high}\textit{ BCR}^{low}\textit{DZ} \times \frac{\%\textit{mCherry}^{low}\textit{ Venus}^{high}\textit{ in BCR}^{low}\textit{ DZ}}{100}
\end{aligned}
$$

Frequencies of stop codons in the total DZs and LZs were calculated similarly, for example the frequency of stop codons in total DZs were calculated as such:

$$\begin{aligned}\textit{frequency of stop codons in DZ} &= \textit{frequency of stop codons in BCR}^{high}\textit{DZ} \times \frac{\%\textit{BCR}^{high}\textit{ in DZ}}{100}\\ &+ \textit{frequency of stop codons in BCR}^{low}\textit{DZ} \times \frac{\%\textit{BCR}^{low}\textit{ in DZ}}{100}\end{aligned}$$

And the frequency of stop codons in the whole GC was therefore calculated:

$$\begin{aligned}\textit{frequency of stop codons in whole GC} &= \textit{frequency of stop codon in DZ} \times \frac{\%\textit{ DZ in whole GC}}{100}\\ &+ \textit{frequency of stop codon in LZ} \times \frac{\%\textit{ LZ in whole GC}}{100}\\ &+ \textit{frequency of stop codon in CXCR4}^{low}\textit{CD83}^{low} \times \frac{\%\textit{ CXCR4}^{low}\textit{CD83}^{low}\textit{in whole GC}}{100}\end{aligned}$$

Estimates of expected premature stop codon frequency

At risk codons

We first consider single nucleotide substitutions. The SW_{HEL} *Ighv* region gene was annotated to determine which codons were at risk of generating stop codons by single point changes at any position. Codons were classified as to whether one point mutation, or two different singular point mutations could terminate the gene. For the former, there is a 1 in 3 chance that a mutation will occur in the stop codon causing position, and a 1 in 3 chance that the mutation will yield a premature stop codon (1 in 9 chance total). The "risk" is doubled (2 in 9) where substitutions in either of 2 different positions can cause stops, or if substitutions of two different nucleotides in the same position could cause stops. For any given gene sequence, the likelihood of a mutation leading to a stop codon is equal to the frequency of "at risk" codons multiplied by 1/9, plus the frequency of codons which are "doubly at risk" multiplied by 2/9.

Enrichment at specific sites

SHM occurs preferentially in complementarity determining regions (CDRs) and following specific motifs. To account for this in a crude manner, we worked with an assumption that mutations were 5 times more likely to occur in CDRs, and 2 times more likely to occur in motif associated regions. We demarked the SW_{HEL} *Ighv* region gene CDRs; and motif containing regions, both within the CDRs and outside the CDRs. The frequency of point mutations expected to occur in a region is equal to the size of that region, multiplied by the enrichment (2 for 2 times, 5 for 5 times) for that region, divided by the sum of sizes of all other regions of the gene, each multiplied by their respective enrichments.

The frequency of SHMs which will cause a premature stop codon in a given region is equal to the likelihood of a point mutation causing a premature stop codon for that region, multiplied by the percentage of SHM that should occur in that region, divided by 100 to give a percentage. The frequency of SHMs expected to cause a stop codon the entire *Ighv* is equal to the sum of the frequencies of SHMs which will cause stop codons in all the constitutive regions of the gene. Using these calculations, the frequency of point mutations which would cause premature stop codons is 6.4%. N.B. the enrichment had a negligible effect, with no enrichment the frequency is 5.8%.

Accounting for established SHM rates per division

SHM is reported to occur at approximately 10^{-3} mutations per division. The SW_{HEL} *Ighv* gene is 393 bases long, and so a cell has a 39.3% chance of acquiring a mutation per division. Therefore, the likelihood of the gene acquiring a premature stop codon by point mutation per division is 6.4 × 39.9/100 = 2.5%.

Risk of insertions and deletions (indels) causing stop codons

All indels that are not units of three (i.e., 2/3 s of indels) are considered to cause eventual stop codons in the gene. It is difficult to estimate the rate of indels, but there is correlative data of numbers of mutations and frequency of deletions (Yeap 2015). By correlating point mutation frequency and deletion frequency, we can estimate the likelihood of a deletion per division. If a Ig gene has a mutational load of X, we assume it has undergone X/0.393 divisions. E.g., if a cell has 20 mutation it is assumed to have completed 51 divisions. Yeap et al. correlated frequencies of point mutations and deletions *in vivo*. If the 40% of cells with 20 SHMs have a deletion, then after 51 divisions 60% of cells do not have a deletion; therefore $(\text{likelihood of not acquiring a deletion per division})^{51} = 0.6$. In this case, the likelihood of not acquiring a deletion per division = 0.99, or 99%. Medium mutation frequency cells, with about 15 mutations, have a 15% frequency of deletion. Low mutation frequency cells, with 5 point mutations, have a frequency of deletion of only 5%. We averaged the likelihood of not acquiring a deletion, and found the chance of not acquiring a deletion per division is 0.994. Therefore, the chance of acquiring a deletion is 0.6%. If one third of those are multiples of three and so do not cause a frameshift mutation, then a cell has a 0.4% chance of acquiring a premature stop codon by deletion per cell division.

Combined risk of induction of premature stop codons by point mutations and indels

The total frequency of SHM causing stop codon is equal to the sum of the likelihood of acquiring a stop codon by point mutation and the likelihood of acquiring a stop codon by deletion. This is 2.5% + 0.4%, so the probability is 2.9%

Frequency of cells with premature stop codons in the dark zone

If cells complete on average two divisions in the DZ, then at a given period 14% will have completed zero divisions, 29% will have completed one division and 57% will have completed two. Those numbers change to 7% (zero), 14% (one), 27% (two) and 54% (three) where the average number of DZ divisions is three. We assume cells with zero divisions to have a have a 0% chance of having acquired a premature stop codon, cells with one division a chance of 2.9% (and as such a 97.1% chance of having not acquired a stop codon per division). The chance of a cell not acquiring a premature stop codon after n divisions is equal to the 0.971^n. Therefore, the chance of acquiring a premature stop codon after n divisions is equal to $1\text{-}0.971^n$. The frequency of cells in the dark zone that carry a premature stop codon is equal to the sum of the proportion of cells which have undergone a given number of divisions multiplied by the likelihood of having acquired a premature stop codon in that number of divisions. For two divisions in the DZ this is 4.1%; for three divisions in the DZ this is 6.4%.

Immunity, Volume *49*

Supplemental Information

Germinal Center B Cells Replace Their Antigen Receptors in Dark Zones and Fail Light Zone Entry when Immunoglobulin Gene Mutations are Damaging

Isabelle Stewart, Daniel Radtke, Bethan Phillips, Simon J. McGowan, and Oliver Bannard

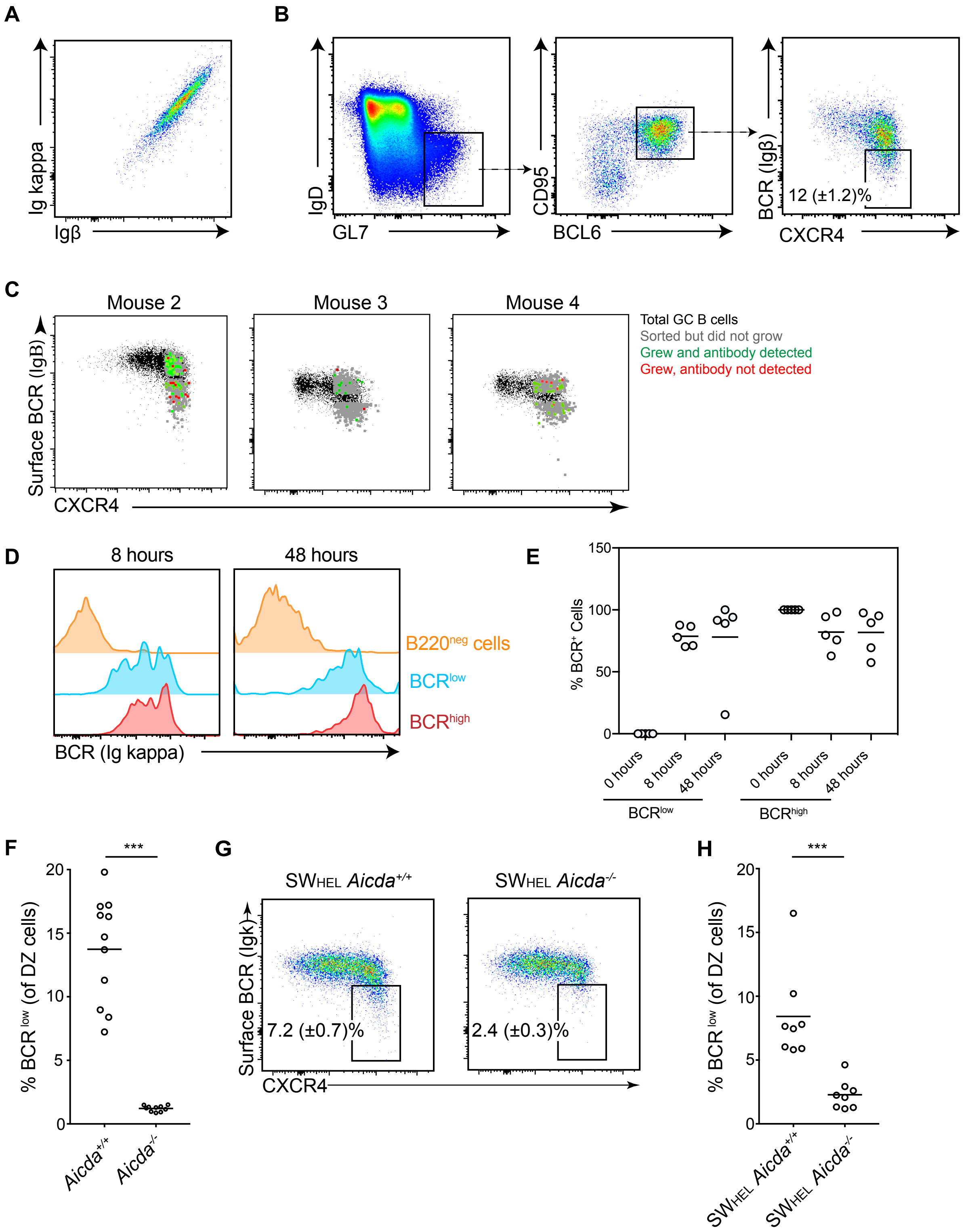
A
Ig kappa
Igβ
B
IgD
GL7
CD95
BCL6
BCR (Igβ)
CXCR4
12 (±1.2)%
C
Mouse 2
Mouse 3
Mouse 4
Surface BCR (IgB)
CXCR4
Total GC B cells
Sorted but did not grow
Grew and antibody detected
Grew, antibody not detected
D
8 hours
48 hours
B220neg cells
BCRlow
BCRhigh
BCR (Ig kappa)
E
% BCR+ Cells
150
100
50
0
0 hours
8 hours
48 hours
0 hours
8 hours
48 hours
BCRlow
BCRhigh
F
***
% BCR low (of DZ cells)
20
15
10
5
0
Aicda+/+
Aicda-/-
G
SWHEL Aicda+/+
SWHEL Aicda-/-
Surface BCR (Igk)
CXCR4
7.2 (±0.7)%
2.4 (±0.3)%
H
***
% BCR low (of DZ cells)
20
15
10
5
0
SWHEL Aicda+/+
SWHEL Aicda-/-

Figure S1 (Related to Figure 1). Characterization of BCR^{low} DZ cells. (A) Surface Igβ and Igκ light chain levels correlate in IgD^{low} CD95^{+} GL7^{+} SWHEL GC B cells (day 7 HEL-OVA/adjuvant immunisation). (B) The CXCR4/BCR staining pattern on BCL6^{+} GC B cells, gated as shown. (C) Results from individual repeat experiments, related to Figures 1F and 1G. Dot plots show BCR and CXCR4 levels on GC B cells at the time of sorting. (D) CXCR4^{high} BCR^{high} and CXCR4^{high} BCR^{low} GC B cells from SRBC immunised mice were sorted for 8 hrs alone, or for 48 hrs under "Nojima" conditions. BCR levels at the end of culture were determined by FACS and compared to that of non-B cells, with data summarised in (E). Labels indicate phenotype at time of sort/plating. (F) Frequencies of *Aicda*$^{+/+}$ and *Aicda*$^{-/-}$ CXCR4^{high} CD83^{low} DZ GC B cells that have low BCR levels in mixed BM chimeric mice on days 7 or 8 of response to SRBC immunisation, related to Figure 1H. (G), (H), Similar assessments were made for co-transferred *Aicda*$^{+/+}$ and *Aicda*$^{-/-}$ SWHEL GC B cells. (A), (B) show representative plot of 7 and 6 mice from 2 experiments, (D) is representative and (E) pooled from 5 mice in 2 experiments, (F) is pooled from 3 experiments and (G)-(H) are from 2 experiments. Numbers in (B), (G) represent mean +/- S.E.M., dots in (E), (F), (H) represent single mice. Analysis was performed using an unpaired two-tailed student's t-test, ***, P <0.001.

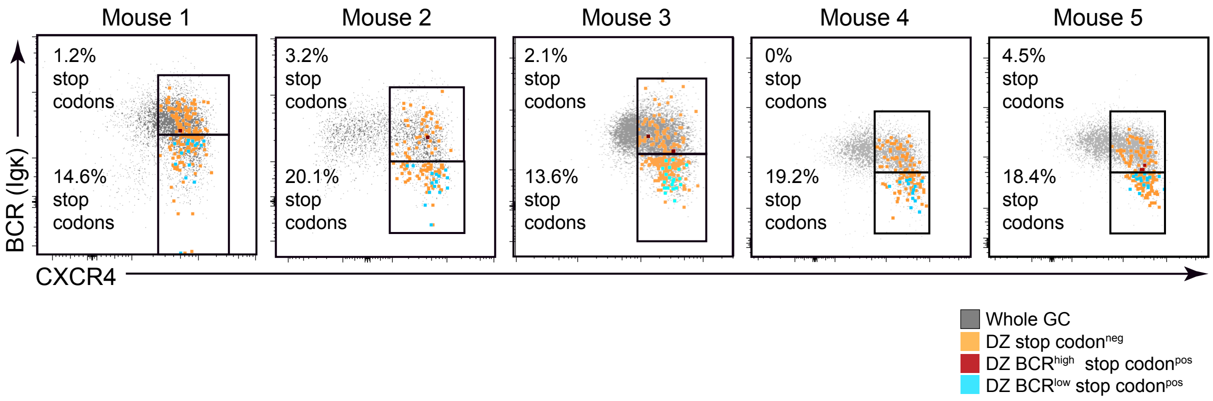

Mouse 1
1.2% stop codons
14.6% stop codons
Mouse 2
3.2% stop codons
20.1% stop codons
Mouse 3
2.1% stop codons
13.6% stop codons
Mouse 4
0% stop codons
19.2% stop codons
Mouse 5
4.5% stop codons
18.4% stop codons
BCR (Igκ)
CXCR4
Whole GC
DZ stop codon^neg
DZ BCR^high stop codon^pos
DZ BCR^low stop codon^pos

Figure S2 (Related to Figure 2). Cells with premature stop codons accumulate in BCR^{low} DZ subsets. Individual repeats of indexed sequenced cells summarised in Figure 2C (with mouse 3 being the same animal as in Figure 2B). Colours indicate presence or absence of *Ighv* region premature stop codons in cells of the indicated phenotype. Frequencies of premature stop codons in BCR^{high} and BCR^{low} DZ gates are shown.

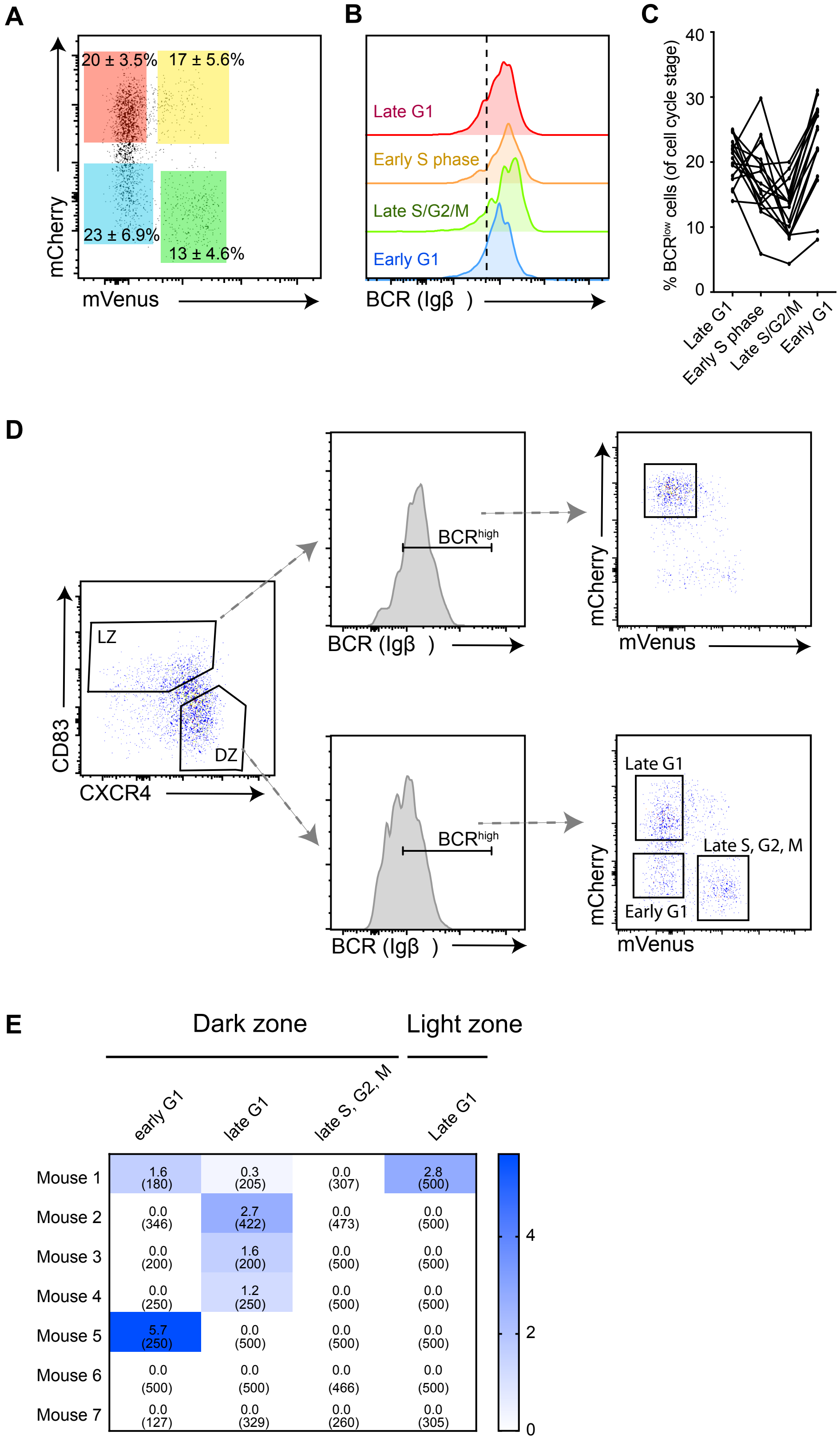
A
20 ± 3.5%
17 ± 5.6%
23 ± 6.9%
13 ± 4.6%
mCherry
mVenus
B
Late G1
Early S phase
Late S/G2/M
Early G1
BCR (Igβ)
C
% BCR^low cells (of cell cycle stage)
40
30
20
10
0
Late G1
Early S phase
Late S/G2/M
Early G1
D
LZ
DZ
CD83
CXCR4
BCR^high
BCR (Igβ)
mCherry
mVenus
Late G1
Late S, G2, M
Early G1
E
Dark zone
Light zone
early G1
late G1
late S, G2, M
Late G1
Mouse 1 1.6 (180) 0.3 (205) 0.0 (307) 2.8 (500)
Mouse 2 0.0 (346) 2.7 (422) 0.0 (473) 0.0 (500)
Mouse 3 0.0 (200) 1.6 (200) 0.0 (500) 0.0 (500)
Mouse 4 0.0 (250) 1.2 (250) 0.0 (500) 0.0 (500)
Mouse 5 5.7 (250) 0.0 (500) 0.0 (500) 0.0 (500)
Mouse 6 0.0 (500) 0.0 (500) 0.0 (466) 0.0 (500)
Mouse 7 0.0 (127) 0.0 (329) 0.0 (260) 0.0 (305)
4
2
0

Figure S3 (Related to Figure 3). BCR^{low} cells are present at all cell cycle stages in the DZ but SHM may occur preferentially in G1. The BCR^{low} state is not restricted to certain cell cycle stages in the dark zone. SWHELxFucci2 DZ (CXCR4^{high} CD83^{low}) GC B cells (IgD^{low} GL7^{+} CD45.1 or 2^{+} or CD95^{+} GL7^{+} CD45.1 or 2^{+}) 8 days after immunisation with HEL-OVA/adjuvant were gated for their cell cycle status (A) and BCR levels determined (B). Dashed line marks BCR^{low} gating. Summary of results from multiple mice and experiments are shown in (C) with each line of connected dots representing results from a single animal. (D) Representative gates used to sort BCR^{high} cells at different cell cycle stages for bulk population based NGS analysis of stop codon presence. (E) The frequency of stop codons detected within each population is shown, with the lower numbers in brackets indicating population sizes sorted from each gate and mouse. Numbers in (A) are means +/- S.D., and (B) is representative of, 13 mice. Six *Aicda*$^{-/-}$ control populations were included in (E) and showed the expected patterns of zero stop codons (not shown).

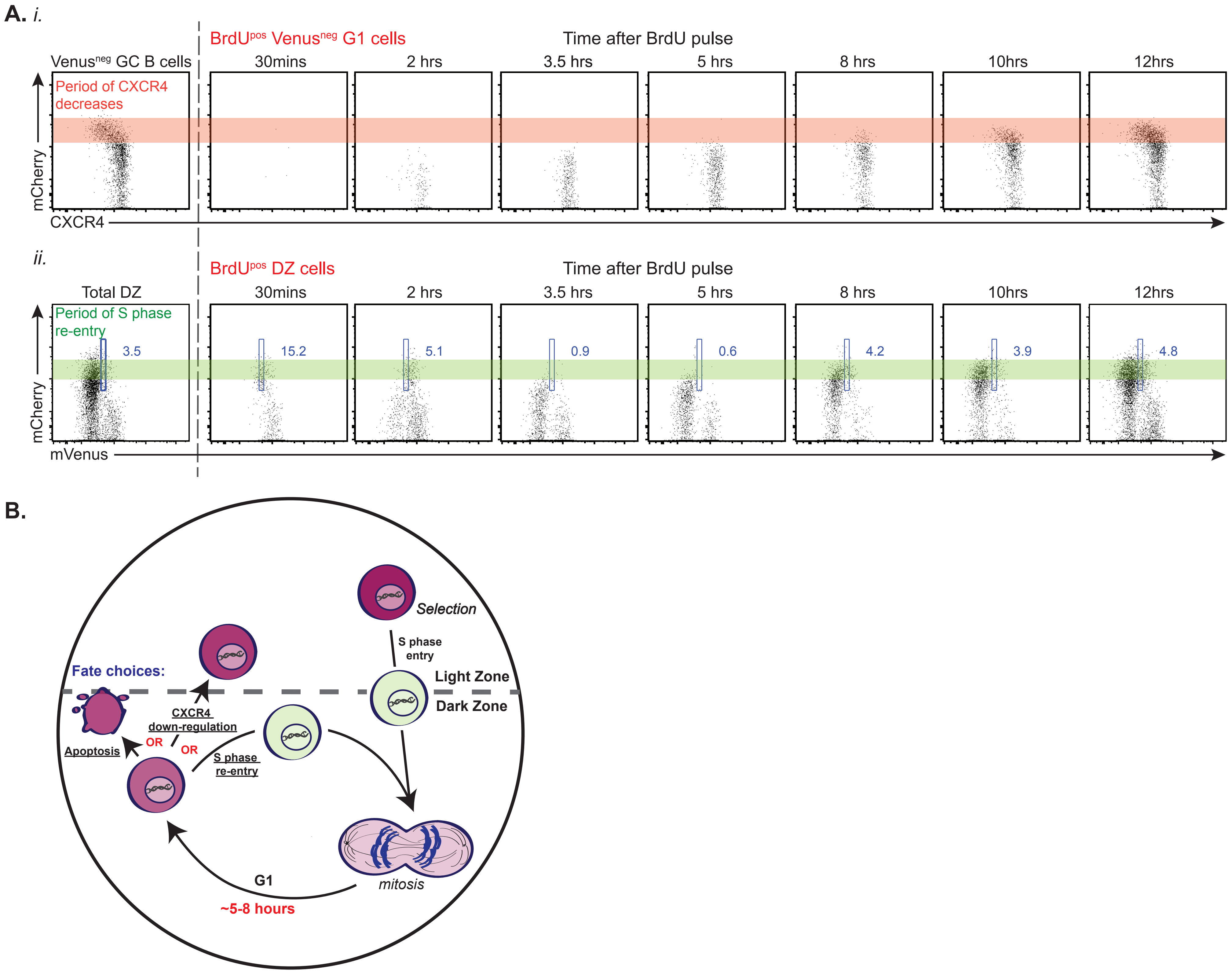
A.
i.
BrdUpos Venusneg G1 cells
Time after BrdU pulse
Venusneg GC B cells
30mins
2 hrs
3.5 hrs
5 hrs
8 hrs
10hrs
12hrs
Period of CXCR4 decreases
mCherry
CXCR4
ii.
BrdUpos DZ cells
Time after BrdU pulse
Total DZ
30mins
2 hrs
3.5 hrs
5 hrs
8 hrs
10hrs
12hrs
Period of S phase re-entry
3.5
15.2
5.1
0.9
0.6
4.2
3.9
4.8
mCherry
mVenus
B.
Selection
S phase entry
Light Zone
Dark Zone
Fate choices:
CXCR4 down-regulation
OR
OR
Apoptosis
S phase re-entry
mitosis
G1
~5-8 hours

Figure S4 (Related to Figure 5). The temporal relationship between G1 sub-stage and S phase re-entry, or CXCR4 reductions, by DZ cells. (A) Fucci2 mice were immunised with SRBCs and received single BrdU injections at the indicated time points before analysis on day 10. Plots to left of dash show whole populations irrespective of whether they are BrdU^{+} or BrdU^{neg}, plots to right of dash show just BrdU^{+} cells. (i) mVenus (G1) GC B cells were gated to determine the timing of surface CXCR4 decreases after the last cellular division. (ii) CXCR4^{high} CD83^{low} DZ cells were gated. Orange (i) and green (ii) shaded areas indicate ranges of mCherry at which CXCR4 level reductions or S phase re-entry occurs. Very early S phase DZ cells are gated (blue rectangles) in (ii). (B) Cartoon illustrating fate decisions in the GC. “Selected” cells enter S phase in the LZ and complete this first division in the DZ before spending a period of approximately 5-8 hrs in G1. Late G1 DZ cells commit to one of three fates; initiation of another cell division, reduce their CXCR4 levels in preparation for migration to the LZ, or death by apoptosis. Data in (A) are representative of 4-5 mice per time point from 2 independent experiments.

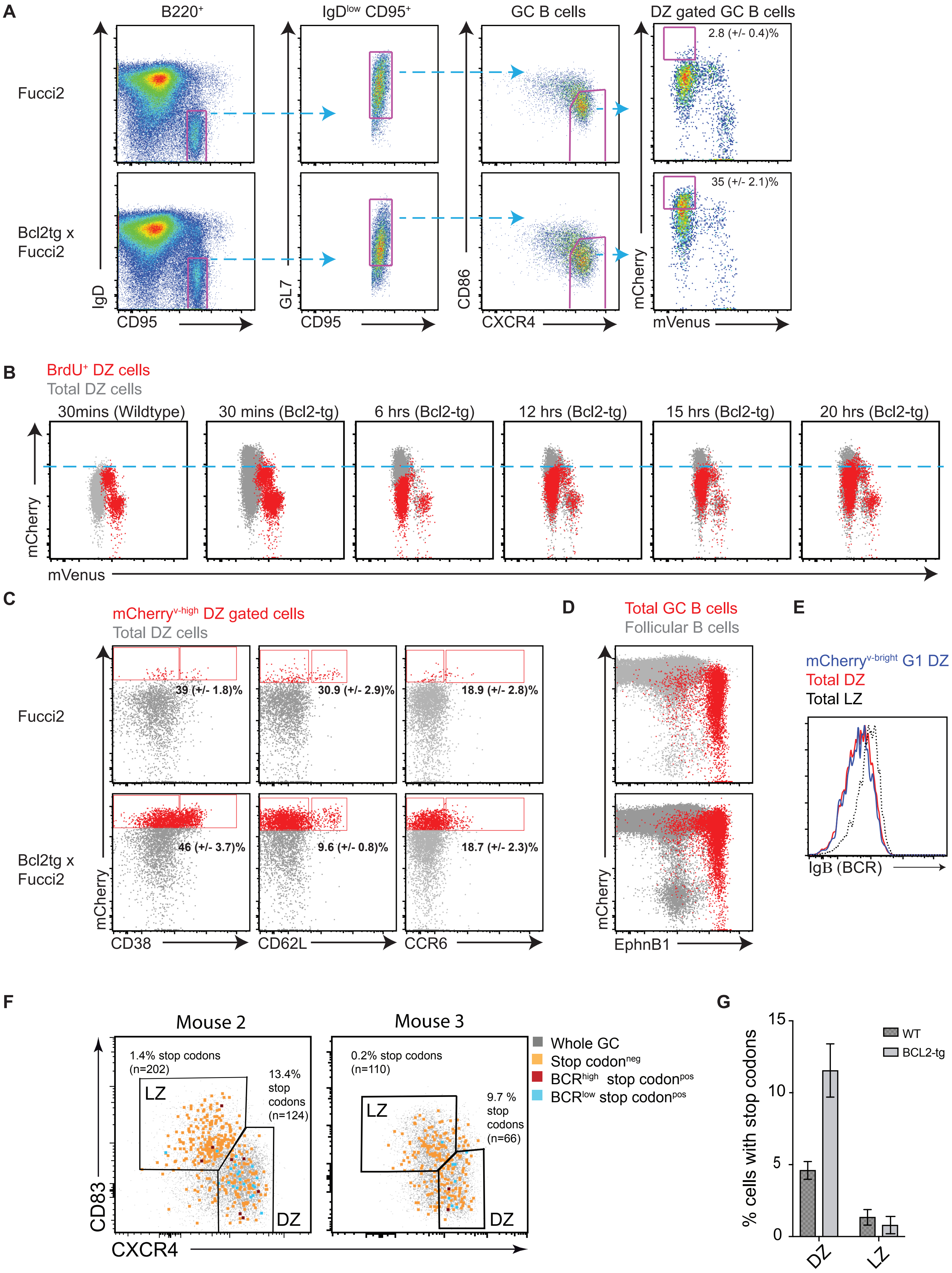

A
B220+
IgDlow CD95+
GC B cells
DZ gated GC B cells
Fucci2
Bcl2tg x
Fucci2
2.8 (+/- 0.4)%
35 (+/- 2.1)%
IgD
CD95
GL7
CD95
CD86
CXCR4
mCherry
mVenus
B
BrdU+ DZ cells
Total DZ cells
30mins (Wildtype)
30 mins (Bcl2-tg)
6 hrs (Bcl2-tg)
12 hrs (Bcl2-tg)
15 hrs (Bcl2-tg)
20 hrs (Bcl2-tg)
mCherry
mVenus
C
mCherryv-high DZ gated cells
Total DZ cells
Fucci2
39 (+/- 1.8)%
30.9 (+/- 2.9)%
18.9 (+/- 2.8)%
Bcl2tg x
Fucci2
46 (+/- 3.7)%
9.6 (+/- 0.8)%
18.7 (+/- 2.3)%
mCherry
CD38
CD62L
CCR6
D
Total GC B cells
Follicular B cells
mCherry
EphnB1
E
mCherryv-bright G1 DZ
Total DZ
Total LZ
IgB (BCR)
F
Mouse 2
Mouse 3
1.4% stop codons
(n=202)
LZ
13.4%
stop
codons
(n=124)
DZ
0.2% stop codons
(n=110)
LZ
9.7 %
stop
codons
(n=66)
DZ
Whole GC
Stop codonneg
BCRhigh stop codonpos
BCRlow stop codonpos
CD83
CXCR4
G
WT
BCL2-tg
% cells with stop codons
15
10
5
0
DZ
LZ

Figure S5 (Related to Figure 6). Aberrant quiescent populations exist in the DZs of Bcl2-tg mice. Splenic GC B cells from SRBC immunised wildtype or Bcl2-tg mice carrying the Fucci2 allele were gated and mCherry levels within DZ cells were determined (A). (B) Mice received single BrdU injections at the indicated time points before analysis. $BrdU^{+}$ DZ cells (red) are overlaid with total DZ cells (grey). The blue dashed bar (equivalent to maximal mCherry levels in WT DZ cells) and is shown for reference. Levels of the various pre-memory markers (C), and the GC and pre-memory associated receptor Ephrin B1 (D), were determined in $mCherry^{v\text{-}bright}$ DZ cells (red) and were overlaid with the total DZ population for reference (grey). Numbers indicate frequency of $mCherry^{v\text{-}bright}$ $mVenus^{neg}$ (red) cells that were positive for indicated protein. (E) BCR levels in GC B populations ($CD38^{low}$ IgD^{low} $GL7^{+}$) of the indicated phenotype. GC DZ/LZ cells were gated using CXCR4 and CD83 for this experiment. (F) Individual repeats of indexed sequenced SWHEL x Bcl2-tg cells that are summarised in Figure 6E. Colours indicate presence or absence of premature stop codons in cells of the indicated phenotype. Frequencies of premature stop codons in DZ and LZ gates are shown, with numbers of cells sequenced shown in brackets. (G) Summary of frequency of stop codons in DZ and LZ of WT (from Figure 4, n=5) and BCL2-tg (n=2) SWHEL GC B cells. Error bars, S.E.M.. Numbers in (A, C, D) are means (+/- S.E.M.) of 6 (WT) and 10 (Bcl2-tg) mice from 2 experiments. Each plot in (B) is from representative of 3-4 mice at each time point from 2 experiments and plots in (E) are representative from 3 experiments each containing 2-3 mice.
